# Revisiting the vanishing refuge model of diversification

**DOI:** 10.3389/fgene.2014.00353

**Published:** 2014-10-22

**Authors:** Roberta Damasceno, Maria L. Strangas, Ana C. Carnaval, Miguel T. Rodrigues, Craig Moritz

**Affiliations:** ^1^Museum of Vertebrate Zoology, Integrative Biology Department, University of California BerkeleyBerkeley, CA, USA; ^2^Departamento de Zoologia, Instituto de Biociências, Universidade de São PauloSão Paulo, Brazil; ^3^Biology Department, The Graduate Center, City University of New YorkNew York, NY, USA; ^4^Biology Department, City College, City University of New YorkNew York, NY, USA; ^5^Research School of Biology, The Australian National UniversityActon, ACT, Australia

**Keywords:** vanishing refuge model, speciation, diversification, phenotypic evolution, habitat stability, niche evolution

## Abstract

Much of the debate around speciation and historical biogeography has focused on the role of stabilizing selection on the physiological (abiotic) niche, emphasizing how isolation and vicariance, when associated with niche conservatism, may drive tropical speciation. Yet, recent re-emphasis on the ecological dimensions of speciation points to a more prominent role of divergent selection in driving genetic, phenotypic, and niche divergence. The vanishing refuge model (VRM), first described by Vanzolini and Williams (1981), describes a process of diversification through climate-driven habitat fragmentation and exposure to new environments, integrating both vicariance and divergent selection. This model suggests that dynamic climates and peripheral isolates can lead to genetic and functional (i.e., ecological and phenotypic) diversity, resulting in sister taxa that occupy contrasting habitats with abutting distributions. Here, we provide predictions for populations undergoing divergence according to the VRM that encompass habitat dynamics, phylogeography, and phenotypic differentiation across populations. Such integrative analyses can, in principle, differentiate the operation of the VRM from other speciation models. We applied these principles to a lizard species, *Coleodactylus meridionalis,* which was used to illustrate the model in the original paper. We incorporate data on inferred historic habitat dynamics, phylogeography and thermal physiology to test for divergence between coastal and inland populations in the Atlantic Forest of Brazil. Environmental and genetic analyses are concordant with divergence through the VRM, yet physiological data are not. We emphasize the importance of multidisciplinary approaches to test this and alternative speciation models while seeking to explain the extraordinarily high genetic and phenotypic diversity of tropical biomes.

## INTRODUCTION

Speciation is a dynamic, multifaceted, and continuous process (Mayr, 1963; de Queiroz, 2007; Mallet, 2007; Berner et al., 2009; Peccoud et al., 2009; Sobel et al., 2010). Much of the debate around speciation and historical biogeography has focused on the role of stabilizing selection on the physiological (abiotic) niche, emphasizing how isolation and vicariance, when associated with niche conservatism, may drive tropical speciation (e.g., Janzen, 1967; Wiens and Graham, 2005). Yet, the recent re-emphasis on the ecological dimensions of speciation points to a more prominent role of divergent selection in driving genetic, phenotypic, and niche divergence (e.g., Endler, 1977; Schluter, 2009; Nosil, 2012). We focus on one opportunity for divergent selection to drive speciation through climate-driven biome shifts. More specifically, we revisit one model of speciation, first referred to by Williams and Vanzolini (1980) and Vanzolini (1981), and formally proposed by Vanzolini and Williams (1981): the vanishing refuge model (VRM). This model predicts vicariance and subsequent divergent selection as habitats change over time. Developed to explain the distribution of sister species in adjacent yet environmentally contrasting biomes, Vanzolini and Williams’ (1981) model states that habitat shifts can lead to genetically and phenotypically divergent species, without invoking mechanisms of divergence with gene flow such as parapatric speciation (e.g., Endler, 1977, 1982):

“Some populations of forest-restricted species may be pre-adapted to life in open formations. If, during the dry part of a climatic cycle, they happen to be confined to a refuge that eventually vanishes, they may, in the process, become completely adapted to open formation conditions and constitute a full ecological variant.”

(Vanzolini and Williams, 1981)

Vanzolini and Williams (1981) proposed the VRM as a variant of allopatric speciation, foreseeing eco-geographic isolation (Sobel et al., 2010), genetic and phenotypic divergence, and, ultimately, speciation, as original forest habitats shrink and disappear. While the VRM builds on the Pleistocene refuge hypothesis (PRH; Haffer, 1969), the two models are clearly distinct. Both the PRH and the VRM stress the geographic setting of allopatric divergence, yet the PRH addresses divergence across isolated patches of similar habitats (e.g., forest refugia), whereas the VRM specifically focuses on divergent evolutionary trajectories across distinct habitat types (continuing forest vs. former forest but now savanna). Speciation and phylogeographic breaks across biomes, as well as eco-phenotypic divergence in response to climate-driven changes in habitat distribution, are uniquely under the domain of the VRM. Yet, the VRM has sometimes been inappropriately linked to speciation across similar (e.g., forest) habitats (e.g., Viljanen, 2009; Wirta, 2009; de Mello Martins, 2011; Prado et al., 2011; de Carvalho et al., 2013).

Revisiting and refining the VRM is relevant for present-day discussions about the drivers of diversification, particularly given the renaissance of ecological and biogeographic thinking in speciation studies, and the new methods used to infer population and biogeographic history. This often neglected model mirrors recent emphasis on the role of ecology and adaptive divergence on speciation processes (Nosil, 2012; Arnegard et al., 2014). Though the VRM has been evoked to explain biogeographical patterns in the tropics (Almeida et al., 2007; Graham et al., 2010; Lim and Sheldon, 2011), it has not, to our knowledge, been tested explicitly. Moreover, the predictions associated with the VRM relative to alternative speciation models, specifically in the context of climate-induced shifts in habitat distributions, have not been clearly defined.

As originally illustrated by Vanzolini and Williams (1981), the VRM describes a process of divergence in which late Pleistocene climatic oscillations led to forest fragmentation, adaptation to new environments, and subsequent speciation. Importantly, however, the model can be used to describe divergence, habitat change, and phenotypic disparity driven by fragmentation of any habitat type, followed by vanishing of the original habitat, and at different time scales.

In this paper, we (1) articulate why and how the VRM contributes to ongoing discussions about the links between diversification and niche evolution; (2) make clear predictions about the expected patterns of genetic structure, phylogenetic relationships, realized niches, and phenotypic disparity resulting from the processes described by the VRM; (3) consider alternative historical processes that can result in diversity patterns similar to those expected under the VRM; (4) discuss multidisciplinary, integrative approaches to test the model; and (5) illustrate an initial test of the predictions of the VRM using new data on the distribution, genetics and physiology of lizards in the Brazilian Atlantic Forest and adjacent dry biomes – the same habitats and taxa used by Vanzolini and Williams (1981) when they first proposed the model.

## THE VRM AND LINKS BETWEEN DIVERSIFICATION AND NICHE EVOLUTION

In general, theory on speciation processes considers the interaction of suppression of gene flow with opportunity for divergent selection and/or genetic drift (Gavrilets, 2003). The VRM assumes that populations of species have become genetically isolated following climate-driven habitat fragmentation of their forest habitats – i.e., allopatric divergence, and that one or more such isolates are then subject to loss of the ancestral (forest) habitat. This combination of isolation and habitat change provides the context for strong differential selection on both biotic and abiotic niche axes, which will enhance the probability of speciation due to rapid build up of genetic incompatibilities (Gavrilets, 2003), ecological barriers to genetically effective dispersal, and potential for correlated responses on mate choice (Nosil, 2012). The PRH also assumes climate-driven fragmentation of forest habitats, but the isolated populations remain in a habitat that is largely similar to that of the ancestral population, therefore being subject to similar selection pressures. In this context, for instance, “mutation-order speciation” posits that similar selection processes operating in isolated populations occupying analogous habitats can nonetheless result in genetic incompatibility because different and incompatible mutations are favored by selection (e.g., Nosil and Flaxman, 2011). This process, which requires long-term isolation, might apply to frequently reported cases of eco-morphologically cryptic speciation among long isolated forest refugia (e.g., Singhal and Moritz, 2013).

Whether the opportunity for speciation under the VRM will be realized depends on the remnant, initially forest-associated, populations remaining viable under divergent selection. Given potentially rapid environmental change, persistence of populations will be enhanced by (i) the presence of standing genetic variation on which selection can act, (ii) plasticity in key traits to buffer the demographic costs of selection, or (iii) a high rate of intrinsic growth relative to the rate of environmental change (Gomulkiewicz and Houle, 2009; Chevin et al., 2010); all “pre-adaptations” to biome shifts. In many cases this will not be possible, resulting in local extinction. Between these two extremes, for instance where forested areas are reduced to small patches in a mosaic of dry habitat types, we might expect selection for broader niches due to spatially varying selection with gene flow, with concomitant change in eco-phenotype, but not a full biome shift. This process might explain phenotypic divergence in peripheral forest refugia in systems that are otherwise phenotypically conservative (Hoskin et al., 2011).

## PREDICTIONS OF THE VRM

Here, we extend the original formulation of the VRM to include specific predictions regarding habitat structure, phylogeography and historical demography that are pertinent to any time period and habitat type (**Table 1A**). Yet we exemplify the predictions with the original conditions illustrated by Vanzolini and Williams (1981).

**Table 1:** (A) Description of the vanishing refuge model and associated predictions.The illustrations on the left were adapted from Vanzolini and Williams (1981); stage descriptions are provided as per the original paper. (B) Possible tests of the various stages described by the model.

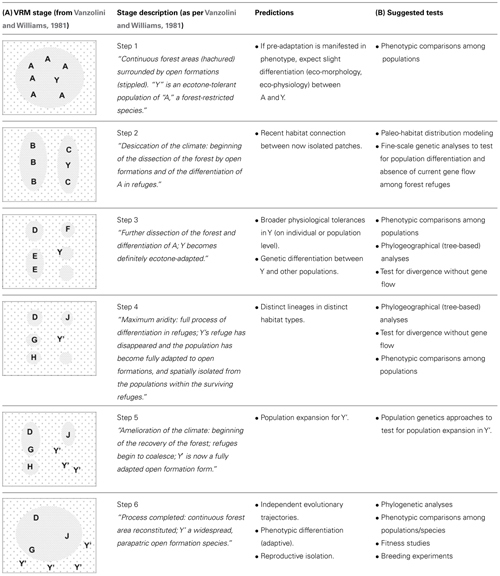

Taxa most amenable to VRM diversification will occur in preferred (ancestral) habitat types (e.g., forest), yet show evidence for “pre-adaptation” (i.e., tolerance) to broader environmental conditions, possibly inferred through natural history observations, for instance through species records in edge or anthropogenic habitats.

Populations of taxa undergoing the initial stages of diversification as described by the VRM will inhabit distinct isolates of suitable habitat. While some populations will remain in more climatically stable (core) areas and under stabilizing selection, others populations will occur in patches (the vanishing refugia) which are being replaced by the surrounding, ecologically distinct matrix. The later will hence be subject to strong directional selection.

At this initial stage, we expect to find evidence of:

(1) Recent habitat connections between core areas and vanishing refugia, followed by habitat fragmentation and near or complete loss of the original preferred habitats; and
(2) Genetic differentiation among populations in core areas and vanishing refugia, due to the cessation of gene flow, and increasing with time since isolation (**Table 1A**, steps 2–6).

Population contraction, associated with the reduction in the size of the preferred habitat and the demographic costs of selection, is possible at this stage – but not a necessary correlate of the process. As natural selection acts upon isolated populations in vanishing refugia, perhaps in combination with genetic drift, these populations will be exposed to the broader range of abiotic conditions found in ecotone environments and are expected to evolve:

(3) Broad physiological tolerances relative to populations in core (stable) areas. This can be detected through:

(a) Inter-individual trait variation (polymorphism), where some individuals in vanishing refugia are more tolerant of the matrix conditions (**Table 1A**, steps 1 and 2);
(b) Greater capacity for plasticity, in terms of either developmental plasticity or (individual) reversible acclimation (**Table 1A**, steps 1 and 2; Angilletta, 2009); or
(c) Adaptation to ecotone conditions, where all individuals are able to tolerate both original habitat and the matrix (**Table 1A**, step 3).

At later stages of the VRM process, we observe that:

(4) Sister lineages occupy distinct *types* of habitat: lineages in core areas occupy ancestral habitat types, whereas lineages evolving in vanishing refugia will have different climatic niches and occupy a distinct (matrix-like) habitat type (**Table 1A**, steps 4–6). In this instance and hereafter, we use the term “lineage” to refer to lineages of individuals (organisms) rather than lineages of genes.

As individuals of the lineage undergoing climatic niche evolution (be it through physiological, morphological, or behavioral evolution) colonize the matrix, we expect to uncover:

(5) Genetic signatures of population expansion in the newly occupied habitat (**Table 1A**, steps 5–6).

Multiple mechanisms may then contribute to:

(6) Pre- or post-mating reproductive isolation between individuals of the ancestral lineages in core habitats relative to those of the newly evolved lineage occupying the matrix, including for instance divergent sexual selection and reinforcement.

In isolation and under the new regime of selective pressures:

(7) The lineage occupying the matrix becomes further differentiated, phenotypically, from those in ancestral habitats. Because the VRM stresses the role of divergent selection, at least some of these phenotypic differences will be adaptive. Whether the physiological tolerances of the diverging lineage will remain broader than that of the ancestral population (or just shift to a different optimum) will depend on the direction of selective pressures in the matrix and hence cannot be predicted.

## ALTERNATIVE PROCESSES THAT LEAD TO SIMILAR PATTERNS OF BIODIVERSITY

The diversification process described by the VRM results in the pattern that originally motivated Vanzolini and Williams (1981): that of sister taxa occupying contrasting habitats with abutting distributions. Yet, this pattern may also be generated by alternative scenarios (Endler, 1982; Doebeli and Dieckmann, 2003; Losos and Glor, 2003), and should not be used as conclusive evidence for the VRM on its own (**Table 2**).

**Table 2:** Differences between the vanishing refuge, parapatric, and peripatric speciation models.

	VRM	Parapatric speciation	Peripatric speciation
Habitat fragmentation at time of divergence	Yes	Not required	Not required
Gene flow during divergence	No	Yes	No
Population contraction at time of divergence	Not required	Little/none	Extreme

The VRM was initially proposed to avoid the assumption of divergence with gene flow, yet a parapatric mode of speciation, in which two populations diverge across an environmental gradient in the presence of continuous gene flow (Mayr, 1963; Endler, 1977; Gavrilets, 2003) could result in the same broad pattern. Under parapatric speciation, the strength of divergent selection must overcome the homogenizing effects of gene flow in order to lead to diversification and speciation (Haldane, 1930; Langerhans et al., 2003; Moore et al., 2007), and thus this process has long been considered biologically difficult. However, several recent studies have found strong evidence of speciation in the presence of gene flow (Niemiller et al., 2008; Pinho and Hey, 2010; Cooke et al., 2012). While parapatric speciation explicitly invokes divergence with gene flow, the VRM suggests divergence in the absence of gene flow.

Peripatric speciation, for instance caused by founder events (Mayr, 1942), may also result in the same geographic patterns of species distribution, gene flow, phylogenetic relationships, genetic isolation, and trait divergence through adaptive speciation as described by the VRM (e.g., Rasner et al., 2004). However, peripatric speciation does not require habitat fragmentation (though is also not incompatible with it), and is expected to result in a severe reduction in population size, which is not necessary under the VRM. The potential for peripatric speciation driven by founder events is highly controversial; though some evidence for founder event speciation has been documented (Templeton, 2008; Balakrishnan and Edwards, 2009; Matute, 2013), many argue that these events are very rare (Coyne and Orr, 2004; Walsh, 2005; Yeung et al., 2011).

## TESTING THE VRM

As with any discussion of refugial dynamics (Gavin et al., 2014) or of speciation (de Queiroz, 2007), multiple forms of evidence are necessary to validate this model. Indeed, no single line of evidence can distinguish between the operation of the VRM and these alternatives (**Table 2**). Given a suitable test system, we suggest integrative testing that combines habitat modeling over climatic fluctuations, genetic analyses, and phenotypic comparisons (**Table 1B**).

### SELECTING A SYSTEM

#### Relevant geographic areas

Regions of high climatic heterogeneity and steep environmental gradients are good candidates for operation of the VRM process because climatic changes, such as those of the Quaternary, can readily lead to habitat deterioration and fragmentation and hence to the isolation and diversification of populations. Extreme differences across habitats can also prevent organisms from experiencing similar microhabitats through behavioral thermoregulation that may otherwise shield them from divergent selection pressures (Gunderson and Leal, 2012). Local environmental analyses (e.g., environmental PCA, Robertson et al., 2001) may help to detect regions with such attributes. The use of correlative habitat models to map stability over time (Graham et al., 2010; Carnaval et al., 2014) should also be used to identify spatial variation in habitat stability and past habitat fragmentation.

#### Candidate taxa

As Vanzolini and Williams (1981) emphasize, exemplars of the VRM process are species or lineages that differ in habitat use relative to their sister taxa and the ancestral state. Appropriate candidate taxa are also expected to have sufficient standing genetic variation and a lack of internal trade-offs to evolve rapidly in response to new selection pressures (Angilletta et al., 2006; Kemp, 2007; Labra et al., 2009). Though difficult to assess directly, these characteristics may be inferred for species with high intra-population variation in key traits or observed habitat preferences. Lability in tolerance traits, as observed across the broader phylogeny (e.g., Grizante et al., 2012), may also provide indirect evidence for evolvability or pre-adaptation.

Generally, we expect that low dispersal organisms will more likely diversify through vanishing refuge processes than will high dispersal species. Because the former may be unable to track habitats through movement or migration (Araújo et al., 2008; Sandel et al., 2011), therefore failing to shift their ranges in response to rapid environmental changes (Walther et al., 2002; Wiens and Graham, 2005; Velo-Anton et al., 2013), these species are more readily exposed to new and harsh selection regimes such as those assumed in the VRM.

### TESTING THE HYPOTHESIS

#### Environmental analyses

A key approach to validating the VRM is through paleo-habitat modeling. By using bioclimatic distribution models to infer the distribution of the inferred ancestral habitat rather than individual species, this approach can reveal past habitat fragmentation (Graham et al., 2010; Carnaval et al., 2014) and highlight disjunct areas with lower paleostability within which VRM processes are predicted. Modeling of individual species would be inappropriate in this context, given that VRM predicts niche evolution and species distribution modeling methods assume niche conservatism. Paleo-habitat modeling can help differentiate VRM processes from parapatric or peripatric speciation, as the latter two do not depend on climate and habitat changes (**Table 2**).

#### Genetic analyses

Molecular data can help identify demographic signatures of the VRM at different stages of the diversification model. Coalescent-based methods (e.g., BPP, Yang and Rannala, 2010, and others cited in Fujita et al., 2012) can test for the existence of independently evolving lineages across habitat patches (**Table 1**, step 2). Population genetic methods (e.g., IMa, Hey and Nielsen, 2007) can verify the occurrence of divergence without gene flow (Hey, 2010) and test for population expansion after divergence (Hey and Nielsen, 2004) as expected under the model. Combining the tools of habitat paleomodeling, coalescent simulations, and statistical phylogeography (Hickerson et al., 2006; Carnaval et al., 2009; Knowles, 2009) one can assess the concordance between the time of habitat fragmentation and divergence, and test alternative hypotheses of responses to past environmental shifts. It is also becoming increasingly feasible to locate the region of origin of population expansions (Lemey et al., 2009; Peter and Slatkin, 2013) – and to test whether ecologically derived taxa originated in a region formally occupied by ancestral habitats. In general, these more advanced, coalescent-based analyses require evidence from 100 to 1000s of independent loci, which are now more accessible thanks to new technological advances. These genetic analyses, evidently, will always be limited by the availability of data from each system (Knowles, 2009).

#### Phenotypic analyses

Phenotypic analyses, in combination with phylogeographic evidence, provide crucial evidence on the process of diversification. Though the VRM may be relevant to a broad range of taxa, we focus on phenotypic traits in terrestrial ectothermic vertebrates due to data availability and relevance to the examples of lizard species originally provided by Vanzolini and Williams (1981). Morphology and thermal physiology in such taxa are central to how organisms respond to changes in habitat, and hence can be particularly informative when testing the VRM. Body shape, for instance, is strongly associated with climate and habitat (Kohlsdorf and Navas, 2012) and affects performance traits (Losos, 2009; da Silva et al., 2014), thus suggesting adaptive significance. Limb length and body size are other labile traits that often evolve when habitats change (Mahler et al., 2010). Because thermal physiology directly influences performance traits for ectotherms, critical temperatures and water loss rates can be particularly informative in tests of the VRM (Angilletta, 2009; Sinervo et al., 2010). Unfortunately, little is known about the heritability and evolvability of these thermal physiological traits outside of model systems such as *Drosophila* (Angilletta, 2009).

### CASE STUDY: TESTING VRM WITH COLEODACTYLUS MERIDIONALIS

We illustrate an initial test of the VRM within one lizard species (*Coleodactylus meridionalis*, Sphaerodactylidae, Gekkota) mentioned in the original VRM paper. Vanzolini and Williams (1981) were impressed by the record of one population of this species in the Caatinga biome (Exú, state of Pernambuco) that contrasted widely with all the other records known at that point (only in forest habitat). Because of *Coleodactylus meridionalis’* potential exposure to divergent selection, they hypothesized that populations of this species could be in the initial stages of speciation under the VRM.

Vanzolini and Williams (1981) also suggested that three other forest lizard species may be diverging according to the VRM: (1) the sphaerodactylid gecko *Gonatodes humeralis*, (2) the dactyloid *Norops brasiliensis* (at the time *Anolis chrysolepis*), and (3) the tropidurid *Plica plica*. The authors suggested that all of them could possibly be “pre-adapted” to tolerate non-forest habitat. *P. plica* is an arboreal species, typical of primary forest and rarely found at forest edges, which shows occasional basking behavior. *G. humeralis* and *N. brasiliensis* show inter-population variation in habitat use, being found in primary forests as well as highly disturbed habitats. Based on distribution and morphological distinctiveness, Vanzolini and Williams (1981) also hypothesized that the skink *Copeoglossum arajara* (at the time *Mabuya arajara*) has completed the speciation process according to the VRM (Williams and Vanzolini, 1980; Vanzolini and Williams, 1981). This suggestion was made because the authors considered *M. arajara* to be fully adapted to open habitats, whereas *Copeoglossum nigropunctatum* (formerly *M. bistriata*), its putative sister species, was restricted to forested environments (despite being able to actively thermoregulate). Our recent field observations (Rodrigues, personal communication, 2014), however, do not support the presumed differences in habitat use between these two species.

To test whether geographically isolated lineages of *Coleodactylus meridionalis* are diverging according to the VRM, we combined novel occurrence data, preliminary phylogeographic analyses, and physiological assays with existing hypotheses about the historical climatic stability of the Northern AF. Because related species are primarily distributed in forest habitats (Geurgas et al., 2008; Gamble et al., 2011), we suggest this to be the ancestral state for *Coleodactylus meridionalis,* with possible VRM divergence into drier and more open formations. Further increasing its likelihood for exposure to novel selection regimes, *Coleodactylus meridionalis*’ small body size (SVL <5 cm) suggests limited dispersal capacity, which may prevent it from tracking shifting forests in periods of rapid climatic and habitat change.

**FIGURE 1:**
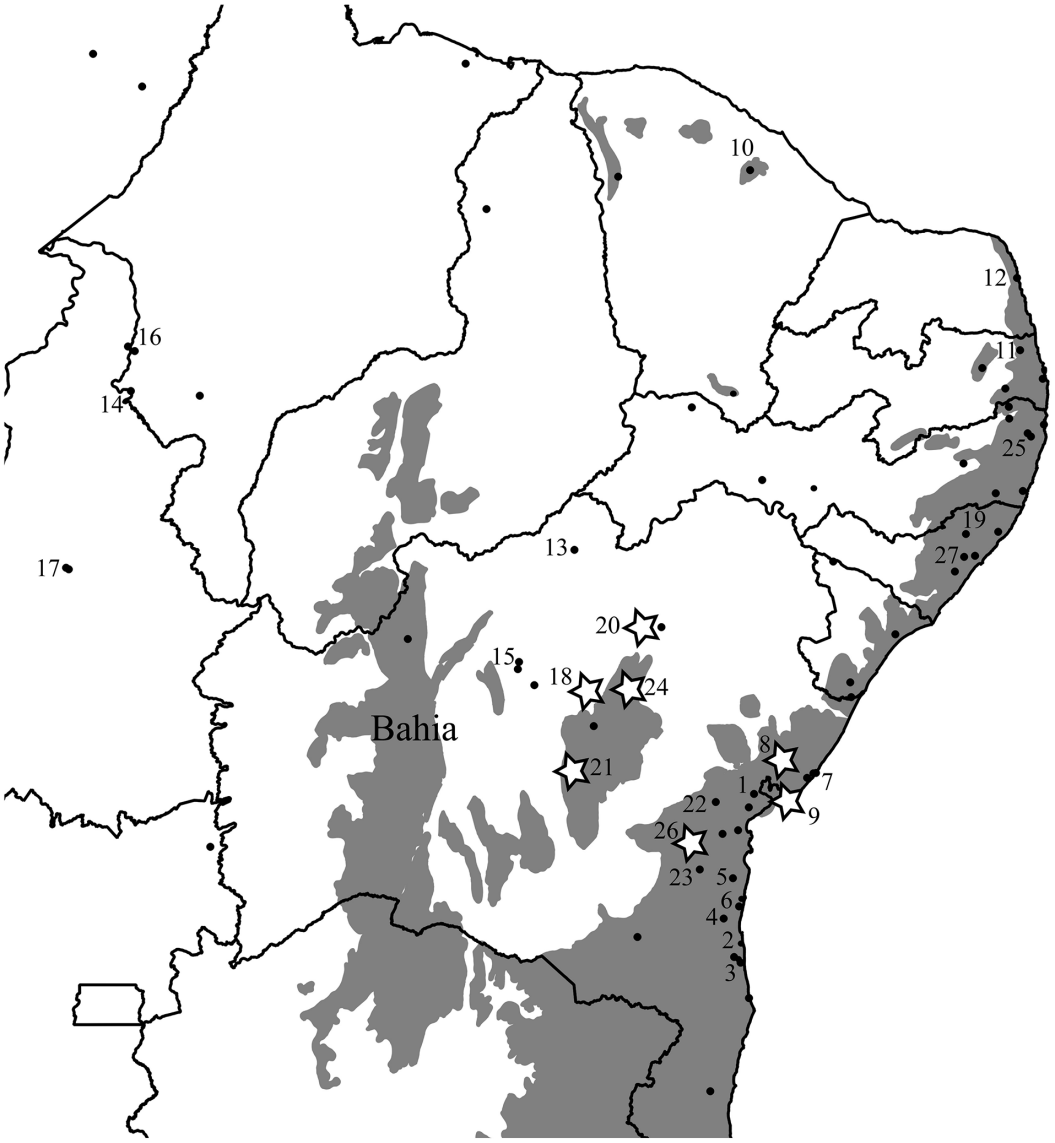
**Original extent of the Brazilian Atlantic Forest biome (in gray; SOS Mata Atlântica and Instituto Nacional de Pesquisas Espaciais, 2012) and the distribution of coastal (core) and isolated (inland) populations of *Coleodactylus meridionalis*.** Numbers correspond to localities included in the genetic analyses and those with stars were included in the physiology dataset. State lines are represented and the State of Bahia is labeled. Only *Coleodactylus natalensis* (morphologically and ecologically distinct clade embedded within *Coleodactylus meridionalis*) is found in locality 12.

**FIGURE 2:**
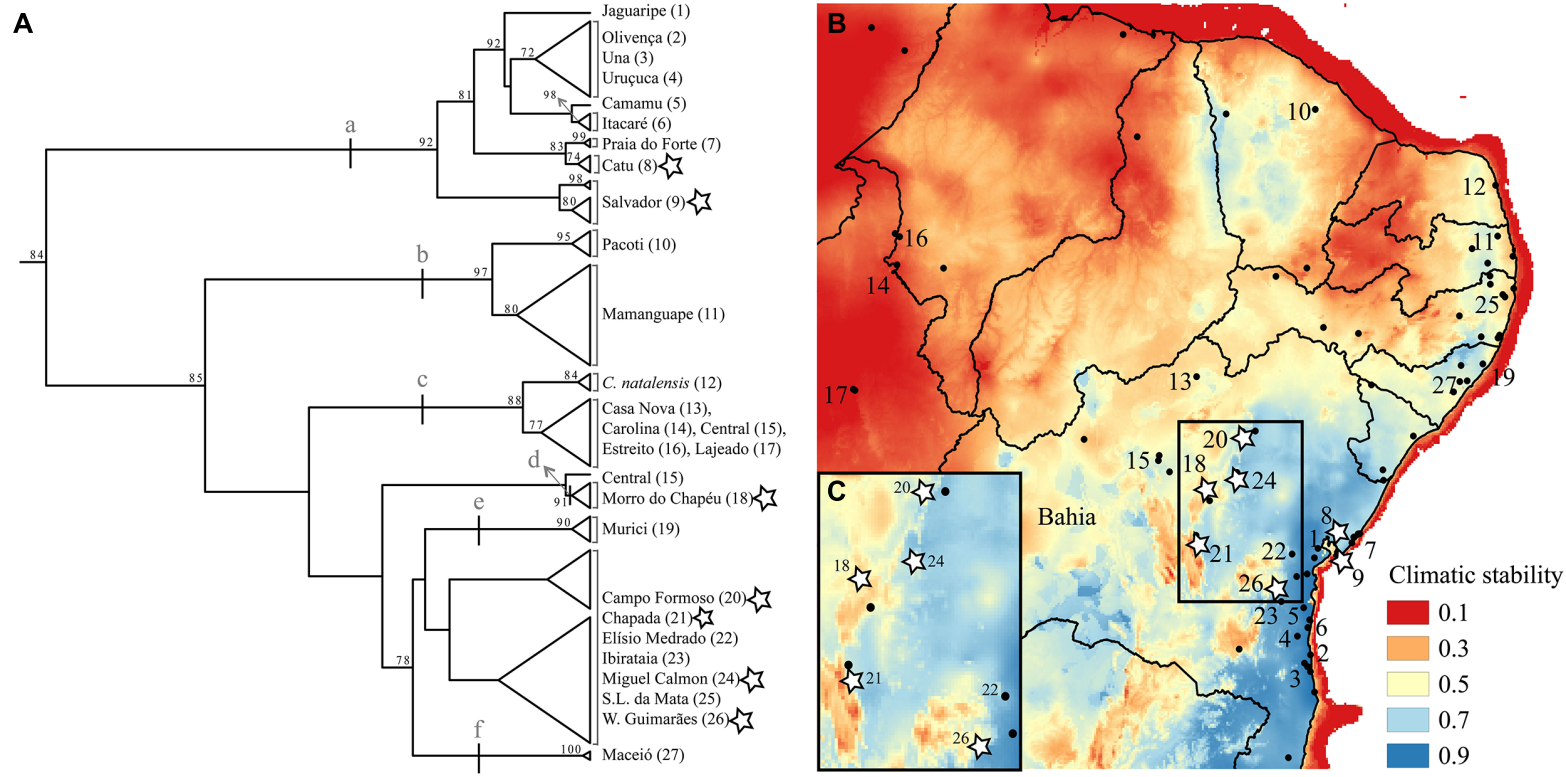
**(A)** mtDNA tree based on the 16S locus using maximum likelihood with bootstrapping. Support values equal or higher than 70 are shown. Letters (a-f) indicate clades described in the results. Names and numbers match the map of Northern Atlantic Forest climatic stability **(B)** over the last glacial cycle (∼120 kya to present-day; data from Carnaval et al., 2014). State lines are represented and the State of Bahia is labeled. Stars mark populations we sampled physiology data for. The insert **(C)** shows in detail climatic stability in the isolates (localities 18, 20, 21, and 24) as well as in one (out of the three) of the coastal localities also examined physiologically (8, 9, and 26).

Our distribution data reveals that this species is indeed restricted to the leaf litter and occurs primarily in the Northern AF, yet is also found in several localities within the much drier Caatinga and Cerrado biomes of Northern and Northeastern Brazil, in addition to the site Vanzolini and Williams (1981) noted at Exú (**Figure 1**). To test for the VRM predictions with *Coleodactylus meridionalis*, we sampled populations in the climatically stable and currently continuous forested area along the Brazilian coast (mostly in Bahia), as well as inland populations in forested areas that have been climatically unstable over the last 120 ky and are currently isolated from coastal AF habitats by the surrounding by Caatinga biome (**Figures 1** and **2**). If VRM mechanisms are in progress, we should find evidence of: (1) recent shifts in the distribution of the AF, (2) recent AF fragmentation, (3) genetic, and (4) phenotypic differentiation between populations in core (stable) forest areas and more unstable, isolated forest patches. We also expect (5) greater acclimation capacity and broader thermal tolerances in lineages occupying historically unstable areas. Although one can expect more within-populations variation (polymorphism) in tolerance traits in unstable areas than in stable areas, our limited sample sizes in some key areas prevents us from testing it statistically.

Two lines of environmental evidence are concordant with a VRM of diversification in this system. Based on correlative paleo-modeling of the Northern AF, developed at 4 ky intervals through a full glacial cycle (120 ky), historical climatic stability of forest habitats has varied throughout this species’ range (Carnaval et al., 2014); some populations have likely been exposed to divergent selection within the last glacial cycle, as habitats shifted in the more climatically unstable areas. Furthermore, present-day occurrence data shows that *Coleodactylus meridionalis* is found in large forested areas along the coast as well as in small, isolated forest patches further inland, with an overall gradient of decreasing historical stability of forest habitat from the coast to the inland regions (**Figures 1** and **2B,C**).

A preliminary phylogeographic analysis based on one mitochondrial locus (16S rDNA), yet covering most of the species distribution (supplementary methods and supplementary Tables 1 and 2), revealed relatively shallow phylogeographic structure within *Coleodactylus meridionalis*. Such low genetic differentiation suggests that, if diverging under the VRM, this system must be in its very early diversification stages (**Figure 2A**). The data nonetheless indicate the existence of a few differentiated clades within this species (bootstrap support >85): (a) a large clade including samples from the climatically stable coastal Bahia, (b) a north coastal clade within a region with low-medium stability, (c) a clade including inland, relatively unstable sites (and *Coleodactylus natalensis* from the coast; see also Geurgas et al., 2008), (d) a distinct lineage comprising individuals from the climatically unstable Morro do Chapéu, and clades including samples from the low-medium stability coastal sites of (e) Murici and (f) Maceió. Of these, the most likely candidate lineage to be diverging under the VRM, given its low climatic stability and geographic location, is that in the high altitude inland site, Morro do Chapéu, that is surrounded by semi-arid Caatinga habitat. In the future, the availability of multi-locus genetic data for *Coleodactylus meridionalis* will enable more rigorous tests of the VRM predictions, improving inferences about the historical demography, gene flow and timing of divergence in this system.

**FIGURE 3:**
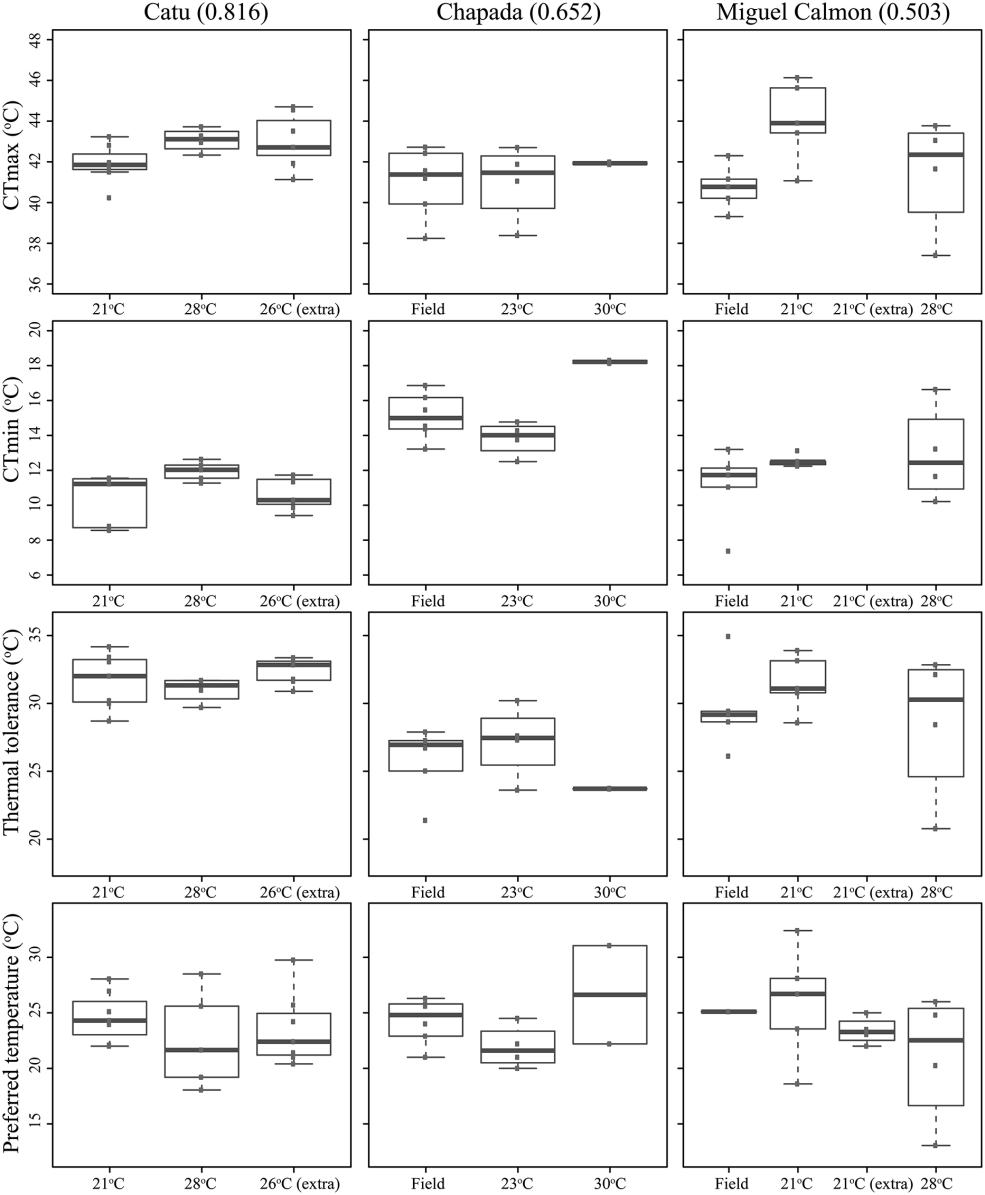
**Critical thermal maximum (CTmax) and minimum (CTmin), thermal tolerances (=CTmax–CTmin), and preferred temperatures of *Coleodactylus meridionalis* populations, right after capture (“Field”) and after acclimation treatments (labeled by the average temperature during each acclimation treatment).** Population order reflects an increase in climatic stability from left to right (stability scores in parentheses).

Existing physiological data, however, do not provide evidence that individuals in historically unstable areas show divergent physiology relative to stable sites. We experimentally measured individual upper and lower critical thermal limits and preferred temperatures in seven localities (CTmin, CTmax, and Tpref, see supplementary methods and supplementary Tables 3 and 4), including Morro do Chapéu, other inland sites, and coastal Bahia, areas with varying levels of inferred paleo-stability for forest (**Figure 2C**). We also assessed short-term reversible acclimation capacity in three localities with varying degrees of climatic stability by conducting experiments immediately after capture as well as after two different acclimation treatments (details in supplementary methods). We nonetheless found no evidence of acclimation capacity in CTmax, CTmin, thermal tolerance (CTmin–CTmax), or thermal preferences (**Table 3**, **Figure 3**), except for a significant shift in CTmin in Chapada (locality 21; **Figure 2C**). Because this result probably reflects the small sample size of the 30°C treatment (two individuals), we avoid over-interpreting it. Nevertheless, if CTmin is actually plastic in Chapada, this would be in opposition to the VRM prediction given the moderate to high stability score of this locality (stability scores presented in supplementary table 2).

**Table 3:** Results of ANOVA with repeated measures to test evidence of acclimation capacity (comparing data after capture and after acclimation treatments).

Response variable	Population	*F*-statistics	*p*-value
CTmin	Chapada	9.993	0.0052
CTmin	Catu	3.613	0.0507
CTmax	Miguel Calmon	3.554	0.0645
CTmax	Catu	2.463	0.1190
Tpref	Chapada	1.973	0.1950
Ttol	Chapada	1.405	0.2940
Ttol	Catu	1.15	0.3430
Tpref	Miguel Calmon	0.848	0.4990
Ttol	Miguel Calmon	0.738	0.5000
Tpref	Catu	0.567	0.5780
CTmin	Miguel Calmon	0.102	0.7580
CTmax	Chapada	0.259	0.7770

**FIGURE 4:**
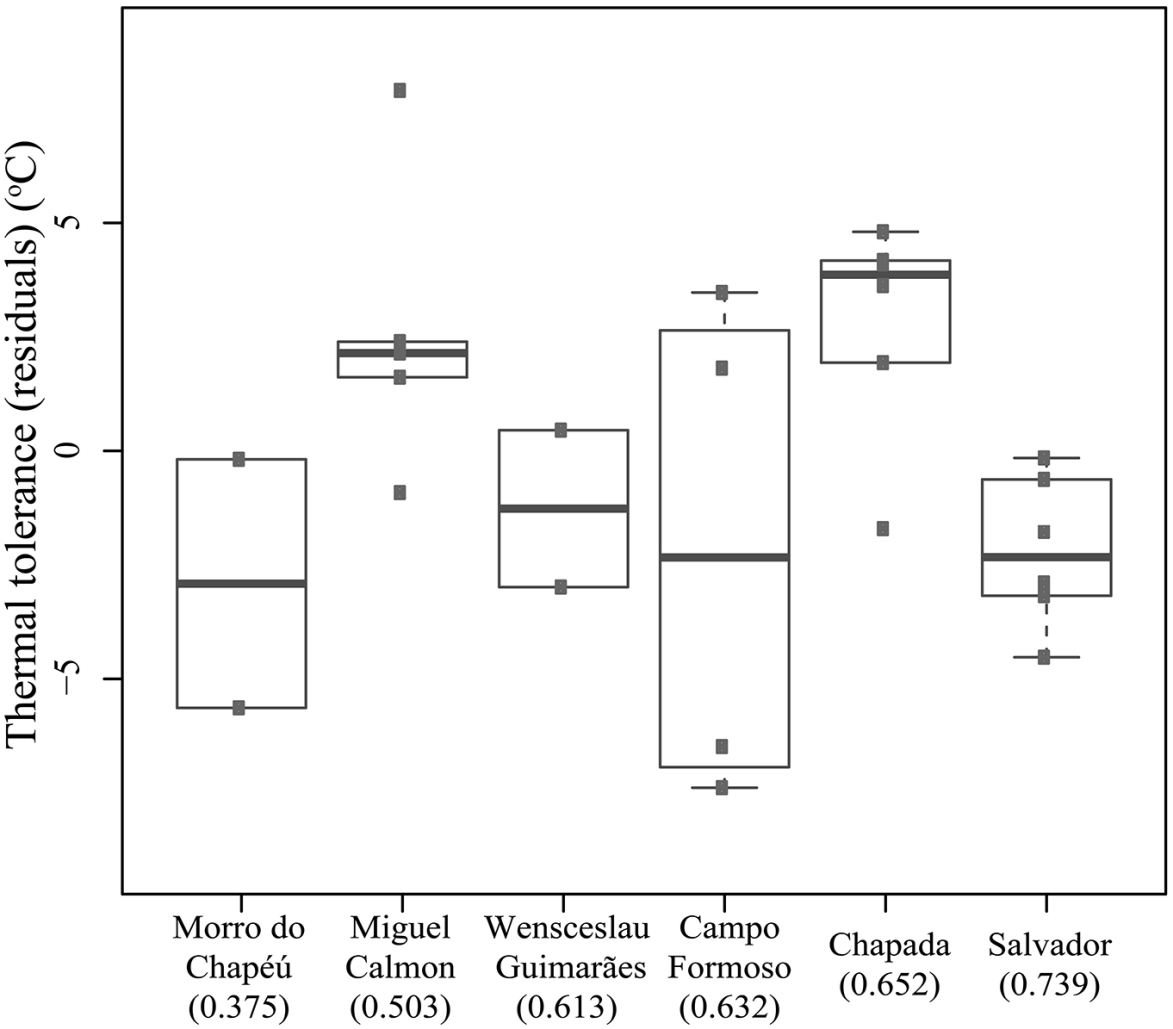
**Thermal tolerance (residuals after correcting for the effect of season) across populations of *Coleodactylus meridionalis* right after capture.** Population order reflects an increase in climatic stability from left to right (stability scores in parentheses).

In contrast to predictions of VRM, we did not detect a correlation between historical habitat stability and thermal tolerance (*R*-squared = 0.015, *p* = 0.554). Tolerance did vary across populations (*F*-statistics = 3.572, *p* = 0.025, corrected for seasonal effects, **Figure 4**), yet the greatest difference (∼5°C) was observed between individuals collected in Salvador (narrower tolerance) relative to those in Chapada (broader tolerance, primarily due to lower CTmin, **Figure 4**). These two sites have high historical climatic stability, yet Salvador is a coastal, lowland site while the Chapada is an inland and higher elevation region where climate is more seasonal (Supplementary Table 3). Together, these results suggest that thermal tolerance in *Coleodactylus meridionalis* may reflect current climate rather than long-term exposure to different levels of climatic fluctuation. Indeed, we found a positive relationship between thermal tolerance (residuals against seasonal effects) and current annual temperature range (*F*-statistics = 4.872, *p* = 0.038). Together, these results suggest that thermal physiology in *Coleodactylus meridionalis* is labile. Whether such correlation represents local adaptation or developmental plasticity is yet to be determined. In contrast to predictions (Khaliq et al., 2014), temperature seasonality does not predict thermal tolerance (residuals; *F*-statistics = 0.4806, *p*-value = 0.495).

It is possible that other phenotypic traits, such as eco-morphology, may have diverged in *Coleodactylus meridionalis* under the VRM. Alternatively, populations in areas with even lower inferred forest stability may be undergoing VRM processes. *Coleodactylus natalensis* (**Figure 1**, clade c) may be one such candidate. This lineage is nested within *Coleodactylus meridionalis*, and may have diverged quite recently Geurgas et al., 2008). It is found in an area with low climatic stability over time, suggesting that it may have been exposed to divergent selective pressures relative to many *Coleodactylus meridionalis* lineages. *Coleodactylus natalensis* is known from only one forested area on a coastal dune system (Capistrano and Freire, 2009, **Figure 1**, locality 12), and is morphologically distinct from sister lineages (Freire, 1999). Some data on thermal physiology exist for the *Coleodactylus natalensis* lineage (de Sousa and Freire, 2011), though these are not yet sufficient to test for VRM processes.

## CONCLUSION

Our case study illustrates how multiple lines of evidence can be combined to identify lineages potentially diverging under the VRM. Recent integrative studies support the view that historic climatic stability promotes the accumulation and *maintenance* of diversity in space and over time (Graham et al., 2006; Carnaval et al., 2009). The vanishing refugia model describes a mechanism by which dynamic climates, hence environmental change and instability, play a key role in *generating* adaptive diversity (Vanzolini and Williams, 1981; Moritz and Carnaval, 2010; Hoskin et al., 2011). Importantly, the diversification process described by the VRM generates high functional diversity, and the resulting taxa are morphologically and/or physiologically distinct. The high genetic diversity observed in stable areas is not expected to show such high morphological and physiological disparity. In addition, the VRM highlights the often overlooked evolutionary potential of peripheral isolates (Moritz et al., 2012). We argue that further identification of lineages and regions undergoing diversification under the VRM will be particularly insightful and relevant to conservation in the face of rapid anthropogenic climate change.

## DATA ACCESSIBILITY

DNA sequences: GenBank accessions KM852739-KM852829.

## AUTHOR CONTRIBUTIONS

Roberta Damasceno and Maria L. Strangas contributed equally to this study. Roberta Damasceno, Maria L. Strangas, Ana C. Carnaval, Craig Moritz, and Miguel T. Rodrigues designed this study. Roberta Damasceno and Miguel T. Rodrigues collected data. Roberta Damasceno performed analyses. Roberta Damasceno, Maria L. Strangas, Ana C. Carnaval, Craig Moritz, and Miguel T. Rodrigues interpreted the data. Roberta Damasceno, Maria L. Strangas, Ana C. Carnaval, and Craig Moritz wrote the manuscript. Miguel T. Rodrigues revised it critically for important intellectual content.

## Conflict of Interest Statement

The authors declare that the research was conducted in the absence of any commercial or financial relationships that could be construed as a potential conflict of interest.

## References

[B1] Almeida F. C. Bonvicino C. R. Cordeiro-Estrela P. (2007). Phylogeny and temporal diversification of *Calomys* (Rodentia, Sigmodontinae): implications for the biogeography of an endemic genus of the open/dry biomes of South America. *Mol. Phylogenet. Evol.* 42 449–466. 10.1016/j.ympev.2006.07.005 16971145

[B2] Angilletta M. J. (2009). *Thermal Adaptation: A Theoretical and Empirical Synthesis*. New York: Oxford University Press. 10.1093/acprof:oso/9780198570875.001.1

[B3] Angilletta M. J. Jr. Bennett A. F. Guderley H. Navas C. A. Seebacher F. Wilson R. S. (2006). Coadaptation: a unifying principle in evolutionary thermal biology. *Physiol. Biochem. Zool.* 79 282–294. 10.1086/499990 16555188

[B4] Araújo M. Nogues-Bravo D. Diniz-Filho J. A. F. Haywood A. M. Valdes P. J. Rahbek C. (2008). Quaternary climate changes explain diversity among reptiles and amphibians. *Ecography* 31 8–15. 10.1111/j.2007.0906-7590.05318.x

[B5] Arnegard M. E. McGee M. D. Matthews B. Marchinko K. B. Conte G. L. Kabir S. (2014). Genetics of ecological divergence during speciation. *Nature* 511 307–311. 10.1038/nature13301 24909991 PMC4149549

[B6] Balakrishnan C. N. Edwards S. V. (2009). Nucleotide variation, linkage disequilibrium and founder-facilitated speciation in wild populations of the zebra finch (*Taeniopygia guttata*). *Genetics* 181 645–660. 10.1534/genetics.108.094250 19047416 PMC2644953

[B7] Berner D. Grandchamp A. C. Hendry A. P. (2009). Variable progress toward ecological speciation in parapatry: stickleback across eight lake-stream transitions. *Evolution* 63 1740–1753. 10.1111/j.1558-5646.2009.00665.x 19228184

[B8] Capistrano M. T. Freire E. M. X. (2009). Utilização de hábitats por *Coleodactylus natalensis* Freire, 1999 (Squamata; Sphaerodactylidae) no Parque Estadual das Dunas do Natal, Rio Grande do Norte. *Publica* 4 48–56

[B9] Carnaval A. C. Hickerson M. J. Haddad C. F. B. Rodrigues M. T. Moritz C. (2009). Stability predicts genetic diversity in the Brazilian Atlantic forest hotspot. *Science* 323 785–789. 10.1126/science.1166955 19197066

[B10] Carnaval A. C. C. Waltari E. Rodrigues M. T. Rosauer D. VanDerWal J. Damasceno R. (2014). Prediction of phylogeographic endemism in an environmentally complex biome. *Proc. R. Soc. B Biol. Sci.* 281 1471–2954. 10.1098/rspb.2014.1461 PMC4150330 25122231

[B11] Chevin L.-M. Lande R. Mace G. M. (2010). Adaptation, plasticity, and extinction in a changing environment: towards a predictive theory. *PLoS Biol.* 8:e1000357. 10.1371/journal.pbio.1000357 PMC2864732 20463950

[B12] Cooke G. M. Chao N. L. Beheregaray L. B. (2012). Divergent natural selection with gene flow along major environmental gradients in Amazonia: insights from genome scans, population genetics and phylogeography of the characin fish *Triportheus albus*. *Mol. Ecol.* 21 2410–2427. 10.1111/j.1365-294X.2012.05540.x 22512735

[B13] Coyne J. A. Orr H. A. (2004). *Speciation.* Sunderland: Sinauer Associates, Inc

[B14] da Silva J. M. Herrel A. Measey G. J. Vanhooydonck B. Tolley K. A. (2014). Linking microhabitat structure, morphology and locomotor performance traits in a recent radiation of dwarf chameleons. *Funct. Ecol.* 28 702–713. 10.1111/1365-2435.12210

[B15] de Carvalho A. L. G. de Britto M. R. Fernandes D. S. (2013). Biogeography of the lizard genus *Tropidurus* Wied-Neuwied, 1825 (Squamata: Tropiduridae): distribution, endemism, and area relationships in South America. *PLoS ONE* 8:e59736. 10.1371/journal.pone.0059736 PMC3602109 23527261

[B16] de Mello Martins F. (2011). Historical biogeography of the Brazilian Atlantic forest and the Carnaval-Moritz model of Pleistocene refugia: what do phylogeographical studies tell us? *Biol. J. Linn. Soc.* 104 499–509. 10.1111/j.1095-8312.2011.01745.x

[B17] de Queiroz K. (2007). Species concepts and species delimitation. *Syst. Biol.* 56 879–886. 10.1080/10635150701701083 18027281

[B18] de Sousa P. A. Freire E. M. (2011). Thermal ecology and thermoregulatory behavior of *Coleodactylus natalensis* (Squamata: Sphaerodactylidae), in a fragment of the Atlantic forest of northeastern, Brazil. *Zoologia (Curitiba)* 28 693–700. 10.1590/S1984-46702011000600001

[B19] Doebeli M. Dieckmann U. (2003). Speciation along environmental gradients. *Nature* 421 259–264. 10.1038/nature01274 12529641

[B20] Endler J. A. (1977). *Geographic Variation, Speciation, and Clines*. Princeton: Princeton University Press 409931

[B21] Endler J. A. (1982). Problems in distinguishing historical from ecological factors in biogeography. *Am. Zool.* 22 441–452. 10.1093/icb/22.2.441

[B22] Freire E. M. X. (1999). Espécie nova de *Coleodactylus* Parker, 1926, das dunas de Natal, Rio Grande do Norte, Brasil, com notas sobre as relações de dicromatismo sexual no gênero (Squamata, Gekkonidae). *Bol. Mus. Nac.* 399 1–14

[B23] Fujita M. K. Leaché A. D. Burbrink F. T. McGuire J. A. Moritz C. (2012). Coalescent-based species delimitation in an integrative taxonomy. *Trends Ecol. Evol.* 27 480–488. 10.1016/j.tree.2012.04.012 22633974

[B24] Gamble T. Daza J. D. Colli G. R. Vitt L. J. Bauer A. M. (2011). A new genus of miniaturized and pug-nosed gecko from South America (Sphaerodactylidae: Gekkota). *Zool. J. Linn. Soc.* 163 1244–1266. 10.1111/j.1096-3642.2011.00741.x 22125341 PMC3223738

[B25] Gavin D. G. Fitzpatrick M. C. Gugger P. F. Heath K. D. Rodríguez-Sánchez F. Dobrowski S. Z. (2014) Climate refugia: joint inference from fossil records, species distribution models, and phylogeography. *New Phytol.* 204 37–54. 10.1111/nph.12929 25039238

[B26] Gavrilets S. (2003). Perspective: models of speciation: what have we learned in 40 years? *Evolution* 57 2197–2215. 10.1111/j.0014-3820.2003.tb00233.x 14628909

[B27] Geurgas S. R. Rodrigues M. T. Moritz C. (2008). The genus *Coleodactylus* (Sphaerodactylinae, Gekkota) revisited: a molecular phylogenetic perspective. *Mol. Phylogenet. Evol.* 49 92–101. 10.1016/j.ympev.2008.05.043 18588990

[B28] Gomulkiewicz R. Houle D. (2009). Demographic and genetic constraints on evolution. *Am. Nat.* 174 E218–E229. 10.1086/645086 19821744

[B29] Graham C. H. Moritz C. Williams S. E. (2006). Habitat history improves prediction of biodiversity in rainforest fauna. *Proc. Natl. Acad. Sci. U.S.A.* 103 632–636. 10.1073/pnas.0505754103 16407139 PMC1334636

[B30] Graham C. H. VanDerWal J. Phillips S. J. Moritz C. Williams S. E. (2010). Dynamic refugia and species persistence: tracking spatial shifts in habitat through time. *Ecography* 33 1062–1069. 10.1111/j.1600-0587.2010.06430.x

[B31] Grizante M. B. Brandt R. Kohlsdorf T. (2012). Evolution of body elongation in gymnophthalmid lizards: relationships with climate. *PLoS ONE* 7:e49772. 10.1371/journal.pone.0049772 PMC3498171 23166767

[B32] Gunderson A. R. Leal M. (2012). Geographic variation in vulnerability to climate warming in a tropical Caribbean lizard. *Funct. Ecol.* 26 783–793. 10.1111/j.1365-2435.2012.01987.x

[B33] Haffer J. (1969). Speciation in amazonian forest birds. *Science* 165 1–7. 10.1126/science.165.3889.131 17834730

[B34] Haldane J. B. S. (1930). A mathematical theory of natural and artificial selection (Part VI, Isolation.). *Math. Proc. Cambridge* 26 220–230. 10.1017/S0305004100015450

[B35] Hey J. (2010). Isolation with migration models for more than two populations. *Mol. Biol. Evol.* 27 905–920. 10.1093/molbev/msp296 19955477 PMC2877539

[B36] Hey J. Nielsen R. (2004). Multilocus methods for estimating population sizes, migration rates and divergence time, with applications to the divergence of *Drosophila pseudoobscura* and *D.* *persimilis. Genetics* 167 747–760. 10.1534/genetics.103.024182 PMC1470901 15238526

[B37] Hey J. Nielsen R. (2007). Integration within the Felsenstein equation for improved Markov chain Monte Carlo methods in population genetics. *Proc. Natl. Acad. Sci. U.S.A.* 104 2785–2790. 10.1073/pnas.0611164104 17301231 PMC1815259

[B38] Hickerson M. J. Stahl E. A. Lessios H. A. (2006). Test for simultaneous divergence using approximate Bayesian computation. *Evolution* 60 2435–2453. 10.1111/j.0014-3820.2006.tb01880.x 17263107

[B39] Hoskin C. J. Tonione M. Higgie M. Mackenzie J. B. Williams S. E. VanDerWal J. (2011). Persistence in peripheral refugia promotes phenotypic divergence and speciation in a rainforest frog. *Am. Nat.* 178 561–578. 10.1086/662164 22030727

[B40] Janzen D. H. (1967). Why mountain passes are higher in the tropics. *Am. Nat.* 101 233–249. 10.1086/282487

[B41] Kemp T. S. (2007). The origin of higher taxa: macroevolutionary processes, and the case of the mammals. *Acta Zool.* 88 3–22. 10.1111/j.1463-6395.2007.00248.x

[B42] Khaliq I. Hof C. Prinzinger R. Böhning-Gease K. Pfenninger M. (2014). Global variation in thermal tolerances and vulnerability of endotherms to climate change. *Proc. R. Soc. B Biol. Sci.* 281 1471–2954. 10.1098/rspb.2014.1097 PMC4100521 25009066

[B43] Knowles L. L. (2009). Statistical phylogeography. *Annu. Rev. Ecol. Evol. Syst.* 40 593–612. 10.1146/annurev.ecolsys.38.091206.095702

[B44] Kohlsdorf T. Navas C. (2012). Evolution of form and function: morphophysiological relationships and locomotor performance in tropidurine lizards. *J. Zool.* 288 41–49. 10.1111/j.1469-7998.2012.00918.x

[B45] Labra A. Pienaar J. Hansen T. F. (2009). Evolution of thermal physiology in *Liolaemus* Lizards: adaptation, phylogenetic inertia, and niche tracking. *Am. Nat.* 174 204–220. 10.1086/600088 19538089

[B46] Langerhans R. B. Layman C. A. Langerhans A. K. Dewitt T. J. (2003). Habitat-associated morphological divergence in two Neotropical fish species. *Biol. J. Linn. Soc.* 80 689–698. 10.1111/j.1095-8312.2003.00266.x

[B47] Lemey P. Rambaut A. Drummond A. J. Suchard M. A. (2009). Bayesian phylogeography finds its roots. *PLoS Comput. Biol.* 5:e1000520. 10.1371/journal.pcbi.1000520 PMC2740835 19779555

[B48] Lim H. C. Sheldon F. H. (2011). Multilocus analysis of the evolutionary dynamics of rainforest bird populations in Southeast Asia. *Mol. Ecol.* 20 3414–3438. 10.1111/j.1365-294X.2011.05190.x 21777318

[B49] Losos J. B. (2009). *Lizards in an Evolutionary Tree: Ecology and Adaptive Radiation of Anoles*. Oakland, CA: University of California Press

[B50] Losos J. B. Glor R. E. (2003). Phylogenetic comparative methods and the geography of speciation. *Trends Ecol. Evol.* 18 220–227. 10.1016/S0169-5347(03)00037-35

[B51] Mahler D. L. Revell L. J. Glor R. E. Losos J. B. (2010). Ecological opportunity and the rate of morphological evolution in the diversification of Greater Antillean Anoles. *Evolution* 64 2731–2745. 10.1111/j.1558-5646.2010.01026.x 20455931

[B52] Mallet J. (2007). Hybrid speciation. *Nature* 446 279–283. 10.1038/nature05706 17361174

[B53] Matute D. R. (2013). The role of founder effects on the evolution of reproductive isolation. *J. Evol. Biol.* 26 2299–2311. 10.1111/jeb.12246 24118666

[B54] Mayr E. (1942). *Systematics and the Origin of Species, from the Viewpoint of a Zoologist*. Cambridge, MA: Harvard University Press

[B55] Mayr E. (1963). *Animal Species and their Evolution.* Cambridge, MA: The Belknap Press of Harvard University Press. 10.4159/harvard.9780674865327

[B56] Moore J. S. Gow J. L. Taylor E. B. Hendry A. P. (2007). Quantifying the constraining influence of gene flow on adaptive divergence in the lake-stream stickleback system. *Evolution* 61 2015–2026. 10.1111/j.1558-5646.2007.00168.x 17683442

[B57] Moritz C. Carnaval A. C. (2010). “Evolutionary biogeography and conservation on a rapidly changing planet: building on darwin’s vision,” in *Darwin* eds Brown W. Fabian A. C. (Cambridge: Cambridge University Press) 135–149

[B58] Moritz C. Langham G. Kearney M. Krockenberger A. VanDerWal J. Williams S. (2012). Integrating phylogeography and physiology reveals divergence of thermal traits between central and peripheral lineages of tropical rainforest lizards. *Philos. Trans. R. Soc. Lond. B Biol. Sci.* 367 1680–1687. 10.1098/rstb.2012.0018 22566675 PMC3350659

[B59] Niemiller M. L. Fitzpatrick B. M. Miller B. T. (2008). Recent divergence with gene flow in Tennessee cave salamanders (Plethodontidae: *Gyrinophilus*) inferred from gene genealogies. *Mol. Ecol.* 17 2258–2275. 10.1111/j.1365-294X.2008.03750.x 18410292

[B60] Nosil P. (2012). *Ecological Speciation.* New York: Oxford University Press

[B61] Nosil P. Flaxman S. M. (2011). Conditions for mutation-order speciation. *Proc. R. Soc. B Biol. Sci.* 278 399–407. 10.1098/rspb.2010.1215 PMC3013408 20702458

[B62] Peccoud J. Ollivier A. Plantegenest M. Simon J.-C. (2009). A continuum of genetic divergence from sympatric host races to species in the pea aphid complex. *Proc. Natl. Acad. Sci. U.S.A.* 106 7495–7500. 10.1073/pnas.0811117106 19380742 PMC2678636

[B63] Peter B. Slatkin M. (2013). Detecting range expansions from genetic data. *Evolution* 67 3274–3289. 10.1111/evo.12202 24152007 PMC4282923

[B64] Pinho C. Hey J. (2010). Divergence with gene flow: models and data. *Annu. Rev. Ecol. Evol. Syst.* 41 215–230. 10.1146/annurev-ecolsys-102209-144644

[B65] Prado C. P. A. Haddad C. F. B. Zamudio K. R. (2011). Cryptic lineages and Pleistocene population expansion in a Brazilian Cerrado frog. *Mol. Ecol.* 21 921–941. 10.1111/j.1365-294X.2011.05409.x 22211375

[B66] Rasner C. A. Yeh P. Eggert L. S. Hunt K. E. Woodruff D. S. Price T. D. (2004). Genetic and morphological evolution following a founder event in the dark-eyed junco, *Junco hyemalis thurberi*. *Mol. Ecol.* 13 671–681. 10.1046/j.1365-294X.2004.02104.x 14871370

[B67] Robertson M. P. Caithness N. Villet M. H. (2001). A PCA-based modelling technique for predicting environmental suitability for organisms from presence records. *Divers. Distrib.* 7 15–27. 10.1046/j.1472-4642.2001.00094.x

[B68] Sandel B. Arge L. Dalsgaard B. Davies R. G. Gaston K. J. Sutherland W. J. (2011). The influence of late Quaternary climate-change velocity on species endemism. *Science* 334 660–664. 10.1126/science.1210173 21979937

[B69] Schluter D. (2009). Evidence for ecological speciation and its alternative. *Science* 323 737–741. 10.1126/science.1160006 19197053

[B70] Sinervo B. Mendez-De-La-Cruz F. Miles D. B. Heulin B. Bastiaans E. Villagrán-Santa Cruz M. (2010). Erosion of lizard diversity by climate change and altered thermal niches. *Science* 328 894–899. 10.1126/science.1184695 20466932

[B71] Singhal S. Moritz C. (2013). Reproductive isolation between phylogeographic lineages scales with divergence. *Proc. R. Soc. B Biol. Sci.* 280:20132246 10.1098/rspb.2013.2246 PMC3813342 24107536

[B72] Sobel J. M. Chen G. F. Watt L. R. Schemske D. W. (2010). The biology of speciation. *Evolution* 64 295–315. 10.1111/j.1558-5646.2009.00877.x 19891628

[B73] SOS Mata Atlântica and Instituto Nacional de Pesquisas Espaciais. (2012). *Atlas dos Remanescentes Florestais da Mata Atlântica, Período de 2010 a 2011*. Available at: http://www.sosmatatlantica.org.br [accessed October 18, 2014]

[B74] Templeton A. R. (2008). The reality and importance of founder speciation in evolution. *Bioessays* 30 470–479. 10.1002/bies.20745 18404703

[B75] Vanzolini P. E. (1981). A quasi-historical approach to the natural history of the differentiation of reptiles in tropical geographic isolates. *Pap. Avulsos Zool.* 34 189–204

[B76] Vanzolini P. E. Williams E. E. (1981). The vanishing refuge: a mechanism for ecogeographic speciation. *Pap. Avulsos Zool. (São Paulo)* 34 251–255

[B77] Velo-Anton G. Parra J. L. Parra-Olea G. Zamudio K. R. (2013). Tracking climate change in a dispersal-limited species: reduced spatial and genetic connectivity in a montane salamander. *Mol. Ecol.* 22 3261–3278. 10.1111/mec.12310 23710831

[B78] Viljanen H. (2009). Life history of *Nanos viettei* (Paulian, 1976) (Coleoptera: Scarabaeidae: Canthonini), a representative of an endemic clade of dung beetles in Madagascar. *Coleopt. Bull.* 63 265–288. 10.1649/1184.1

[B79] Walsh H. E. (2005). A test of founder effect speciation using multiple loci in the Auklets (*Aethia* spp.). *Genetics* 171 1885–1894. 10.1534/genetics.105.043380 16143621 PMC1456112

[B80] Walther G.-R. Post E. Convey P. Menzel A. Parmesan C. Beebee T. J. (2002). Ecological responses to recent climate change. *Nature* 416 389–395. 10.1038/416389a 11919621

[B81] Wiens J. J. Graham C. H. (2005). Niche conservatism: integrating evolution, ecology, and conservation biology. *Annu. Rev. Ecol. Evol. Syst.* 36 519–539. 10.1146/annurev.ecolsys.36.102803.095431

[B82] Williams E. E. Vanzolini P. E. (1980). Notes and biogeographic comments on anoles from Brasil. *Pap. Avulsos Zool. (Sao Paulo)* 346 99–108

[B83] Wirta H. (2009). Complex phylogeographical patterns, introgression and cryptic species in a lineage of Malagasy dung beetles (Coleoptera: Scarabaeidae). *Biol. J. Linn. Soc.* 96 942–955. 10.1111/j.1095-8312.2008.01156.x

[B84] Yang Z. Rannala B. (2010). Bayesian species delimitation using multilocus sequence data. *Proc. Natl. Acad. Sci. U.S.A.* 107 9264–9269. 10.1073/pnas.0913022107 20439743 PMC2889046

[B85] Yeung C. K. Tsai P.-W. Chesser R. T. Lin R.-C. Yao C.-T. Tian X.-H. (2011). Testing founder effect speciation: divergence population genetics of the spoonbills *Platalea regia* and *Pl. minor* (Threskiornithidae, Aves). *Mol. Biol. Evol.* 28 473–482. 10.1093/molbev/msq210 20705906

