# Supplementary material for: Revisiting the vanishing refuge model of diversification
Source: Front Genet. 2014 Oct 22;5:353. doi: 10.3389/fgene.2014.00353 (PMC4205810; doi:10.3389/fgene.2014.00353)
Supplement: Supplementary file 1 [file Data_Sheet_1.DOCX]

***Supplementary Material***

**Revisiting the vanishing refuge model of diversification**

**Roberta Damasceno^*1,2^, Maria L. Strangas^3^, Ana C. Carnaval^3, 4^, Miguel Trefaut Rodrigues^2^, and Craig Moritz^1, 5^**

^1^Museum of Vertebrate Zoology, Integrative Biology Department, University of California, Berkeley, CA, USA  ^2^Departamento de Zoologia, Instituto de Biociências, Universidade de São Paulo, São Paulo, Brazil

^3^Biology Department, Graduate Center, City University of New York, New York, NY, USA

^4^Biology Department, City College of the City University of New York, New York, NY, USA  ^5^Research School of Biology, The Australian National University, Acton, Australia

***Correspondence:**

Dr. Roberta Damasceno

Universidade de São Paulo

Instituto de Biociências

Departamento de Zoologia

Rua do Matão, travessa 14, n 101

São Paulo, 05422-970, Brazil

rpdama@gmail.br

1. **Supplementary Methods**

**Genetic data and analyses**

We sequenced 599 bp of one mitochondrial DNA locus (16S) for 87 individuals of *Coleodactylus meridionalis* and one *C. natalensis* (Supplementary table 1), sampling (27 localities) a gradient of climatic stability and most of *C. meridionalis*’ distribution. We (1) extracted genomic DNA from tissue (liver/tail) samples using high-salt method (Aljanabi and Martinez, 1997), (2) amplified DNA with polymerase chain reaction (PCR) essays, (3) used ABI BigDye Terminator Kit version 3.1 (Applied Biosystems, Foster City, CA) to perform cycle sequencing reactions in both forward and reverse directions, (4) cleaned those products using the ethanol precipitation protocol or Sephadex columns, (5) analyzed the sequences by automated sequencer (ABI 3730, Applied Biosystems, Forest City, CA), and (6) used Geneious Pro v.5.6.5 (Biometters Ltd.) to edit chromatograms and align sequences. We used the primers 16SF and 16SR (Whiting *et al.*, 2003) and the PCR profile was: 94°C (5 min), 35 cycles at 94°C (40 s), 60°C (40 s), 72°C (40 s), 72°C (7 min). Sequences are available on GenBank (accession numbers provided upon acceptance).

We gathered sequence data for 6 taxa from GenBank, including *Coleodactylus natalensis* (2 sequences), *C. brachystoma* (3), *C. septentrionalis* (6), *Chatogekko amazonicus* (2), *Gonatodes humeralis* (1), *Lepidoblepharis xanthostigma* (1), *Pseudogonatodes guianensis* (1) (Supplementary table 2). We used RAxML v. 7.2.8 (Stamatakis, 2006) to construct maximum likelihood trees with GTR-GAMMA model, 1,000 rapid bootstrap inferences and thorough maximum likelihood search. We rooted the tree with *Chatogekko amazonicus*, following previous molecular phylogenetic analyses (Gamble *et al.*, 2011; Geurgas *et al.*, 2008). We performed three RAxML independent runs and inspected concordance in topology and node support.

**Phenotypic analyses – thermal physiology experiments and analyses**

We experimentally measured upper and lower critical thermal limits (CTmax and CTmin) and preferred temperatures (Tpref), immediately after capture and after acclimation treatments in *Coleodactylus meridionalis* individuals from several localities, sampling a gradient of climatic stability, both at the center of the distribution and in geographic isolates (Fig. 1). Measurements immediately after capture are interpreted as representative of the field performances and give insight about the differences among populations resultant from genetic and environmental factors, whereas measurements after acclimation to similar conditions intend to assess/remove the effects of reversible plasticity in thermal physiology. We tested CTs in 34 individuals from seven localities (Campo Formoso, Catu, Chapada, Miguel Calmon, Morro do Chapéu, Salvador, and Wenceslau Guimarães), and tested preferred temperatures in 24 individuals from five of those localities (excluding Campo Formoso and Miguel Calmon, Supplementary table 3).

CTmax and CTmin are indices that represent the points at which locomotory response is compromised yet the lizards will recover (Cowles and Bogert, 1944). They were measured by heating up or cooling down the lizards until righting response was lost (Brooks and Sassaman, 1965). For each experiment, a single animal was placed in a deli cup where a thermocouple (type K or T) was attached so its tip was upright inside the cup. The deli cup was placed close to a heating pad (for CTmax) or inside a mini freezer (for CTmin) and righting response was tested by gently displacing the lizard from its normal resting position, flapping the deli cup so the animal would rest in its back and touch the thermocouple. Rate of temperature change was kept between 0.5 and 0.75 ^o^C/minute, during experiments. After reaching CT, the animal was brought back to a comfortable temperature. There was an interval of at least 12 hours between experiments with the same individual. We calculated thermal tolerance (Ttol) as the difference between CTmax and CTmin.

We created thermal gradients where lizards were allowed to move freely between ~10 and ~45^o^C while body temperatures were recorded. Gradient boxes consisted of plastic boxes (90 x 25 x 20 cm) and temperatures were maintained by positioning heat lamps and ice packs at either end. Body temperatures were recorded every minute for a period of 75 min using an infrared (non-contact) thermometer EXTECH model 42540a (FLIR Commercial Systems, Nashua, USA) positioned as close as possible to the dorso of the animal. We discarded measurements from the initial 15 minutes as lizards adjusted to experimental conditions. Preferred temperature (Tpref) was considered to be the median body temperature during the remaining time. No individuals died during the experiments.

We tested for differences in field Ttol among localities, using ANCOVA and Tukey-Kramer HSD tests, R functions “aov” and “TukeyHSD”, controlling for the effects of season (summer and winter). We tested for correlation between Ttol and climatic stability using the R function “lm”, and the residuals of Ttol after removing the effect of season (stability values and residuals are presented in Supplementary table 3). Climatic stability represents how suitable the climate has been, in a pixel, in the last glacial cycle for the occurrence of the Northern Atlantic Forest (data from Carnaval et al. 2014). A pixel with high stability score is interpret as showing a suitable climate for forest over time and therefore stable. Pixels with dynamic climate show lower stability. In order to test if thermal physiology is responding to current climate (using WorldClim data, Hijmans *et al.* 2005) we also tested for correlation between the residuals of Ttol after removing the effect of season and temperature seasonality (=BIO4, measured as temperature standard deviation over the year) and annual temperature range (=BIO5 – BIO6, the difference between maximum temperature in the warmest month and minimum temperature in the coldest month).

We submitted individuals of three localities (Catu, Chapada, and Miguel Calmon) to two acclimation treatments (“cool” and “warm”). During the acclimation periods, we kept lizards, for 11-18 days, under nearly constant temperatures that were similar to those conditions experienced in the field (Supplementary table 4). To minimize the effects of the order of acclimation treatments, we randomly assigned half the individuals from each locality to one of the two treatments (“cool or “warm”) during the first acclimation period, and later switched individuals to the other treatment for a second acclimation period. When in captivity, animals were kept in individual cages at all times. Animals were offered water every day and fed mealworms every 3 days. We did not measure any thermal physiology traits right after capture in individuals from Catu. For unanticipated logistic reasons, individuals from Catu and Miguel Calmon were subject to additional periods in captivity. Those periods were considered extra acclimation treatments because the duration was not consistent with the planned acclimation periods (Supplementary table 4). To test acclimation capacity in thermal traits, we compared CTmax, CTmin, Ttol, and Tpref in the field and after the acclimation periods for each locality, using analyses of covariance (ANCOVA) with repeated measures, R functions “make.rm” and “aov”. The number of individuals per locality in each treatment varies because some animals died in captivity.

1. **Supplementary Figures and Tables**

## Supplementary Tables

**Supplementary Table 1.** Locality data of samples of *Coleodactylus meridionalis*, *C. natalensis* (Natal), and *C. brachystoma* (São Desidério) used in this study and in the genetic dataset. Locality numbers correspond to those in Figure 1.

| Sample | Locality | Locality number | Latitude | Longitude | GenBank ID |
| --- | --- | --- | --- | --- | --- |
| MZUSP78383 | Água Preta |  | -8.7 | -35.517 |  |
| MZUDP66106 | Areia |  | -6.96747 | -35.70382 |  |
| MTR15586 | Barra do Choça |  | -14.83873 | -40.47752 |  |
| MTR09881 | Barreirinha |  | -2.75756 | -42.85563 |  |
| MZUSP7687 | Buritirama |  | -10.71583 | -43.65619 |  |
| MZUSP56539 | Camaçari |  | -12.63698 | -38.12898 |  |
| MZUSP65702 | Camaçari |  | -12.75 | -38.167 |  |
| PEU295 | Camamu | 5 | -14.02489 | -39.15516 | KM852759 |
| MTR24520 | Campo Formoso | 20 | -10.5628 | -40.40886 | KM852739 |
| MTR24521 | Campo Formoso | 20 | -10.5628 | -40.40886 | KM852740 |
| MTR09770 | Canavieiras |  | -15.683 | -38.933 |  |
| MPEG22243 | Caninde de São Francisco |  | -9.645 | -37.767 |  |
| MZUSP49267 | Carmópolis |  | -10.65096 | -36.9072 |  |
| ESTR00112 | Carolina |  | -7.286 | -47.49 | KM852756 |
| ESTR00349 | Carolina |  | -7.286 | -47.49 | KM852816 |
| ESTR0973 | Carolina |  | -7.286 | -47.49 | KM852821 |
| MZUSP66046 | Caruaru |  | -8.28834 | -35.96385 |  |
| MTR18110 | Casa Nova | 13 | -9.483 | -41.35 | KM852754 |
| MTR18114 | Casa Nova | 13 | -9.483 | -41.35 | KM852775 |

| C12P61 | Catu | 8 | -12.451 | -38.40647 | KM852788 |
| --- | --- | --- | --- | --- | --- |
| C3P21 | Catu | 8 | -12.41133 | -38.47897 | KM852824 |
| P69C12 | Catu | 8 | -12.451 | -38.40647 | KM852792 |
| MTR09882 | Central | 15 | -11.133 | -42.133 | KM852764 |
| MTR09884 | Central | 15 | -11.133 | -42.133 | KM852745 |
| MTR10360 | Central | 15 | -11.133 | -42.133 | KM852741 |
| MTR19942 | Chapada | 21 | -12.7675 | -41.31806 |  |
| MTR19944 | Chapada | 21 | -12.76694 | -41.31778 | KM852785 |
| MZUSP51686 | Chapada | 21 | -7.34915 | -39.08917 |  |
| MTR19913 | Chapada | 21 | -12.60186 | -41.35967 | KM852749 |
| MTR19914 | Chapada | 21 | -12.60186 | -41.35967 | KM852771 |
| RPD133 | Chapada | 21 | -12.60186 | -41.35967 | KM852747 |
| MTR11860 | Elisío Medrado | 22 | -12.97046 | -39.39269 | KM852804 |
| MTR18421 | Elisío Medrado | 22 | -12.97046 | -39.39269 | KM852752 |
| MTR18422 | Elisío Medrado | 22 | -12.97046 | -39.39269 | KM852751 |
| ESTR0549 | Estreito | 16 | -6.733 | -47.436 | KM852757 |
| MZUSP49149 | Exú |  | -7.512 | -39.724 |  |
| GSK 879 | Ibiapaba |  | -4.32021 | -40.7443 |  |
| MTR16651 | Ibirataia | 23 | -13.90441 | -39.614 | KM852803 |
| MTR16652 | Ibirataia | 23 | -13.90441 | -39.614 | KM852755 |
| MZUSP78351 | Ilha de Itamaracá |  | -7.75 | -34.85 |  |
| PEU464 | Ilhéus |  | -15.19305 | -39.0495 |  |
| MZUSP45755 | Inajá |  | -8.5167 | -38.75 |  |
| MZUSP65906 | Inajá |  | -8.633 | -38.0333 |  |
| MNRJ15032 | Indiaroba |  | -11.52013 | -37.5181 |  |
| TGIP | Ipixúna do Pará |  | -2.622 | -48.013 |  |
| MZUSP36707 | Irecê |  | -11.35442 | -41.90449 |  |
| MTR11794 | Itacaré | 6 | -14.41522 | -39.07039 | KM852819 |
| MTR16656 | Itacaré | 6 | -14.41522 | -39.07039 | KM852802 |
| MTR16657 | Itacaré | 6 | -14.41522 | -39.07039 | KM852766 |
| MZUSP11794 | Itacaré | 6 | -14.263 | -39.033 |  |
| PEU087 | Jaguaripe | 1 | -13.04446 | -38.9354 | KM852798 |
| MZUSP59045 | João Pessoa |  | -7.117 | -34.867 |  |
| MPEG22261 | José de Freitas |  | -4.769 | -42.567 |  |
| MZUSP89286 | Jussara |  | -11.03333 | -42.11667 |  |
| MRT6855 | Lajeado | 17 | -9.7523 | -48.349 | KM852820 |
| MRT7086 | Lajeado | 17 | -9.751 | -48.354 |  |
| MTR17819 | Maceió | 27 | -9.56556 | -35.80389 | KM852801 |
| MTR17820 | Maceió | 27 | -9.56556 | -35.80389 | KM852781 |

| MTR9759 | Mamanguape | 11 | -6.72091 | -35.17918 | KM852769 |
| --- | --- | --- | --- | --- | --- |
| MTR9760 | Mamanguape | 11 | -6.72091 | -35.17918 | KM852768 |
| MTR9761 | Mamanguape | 11 | -6.72091 | -35.17918 | KM852787 |
| MTR9762 | Mamanguape | 11 | -6.72091 | -35.17918 | KM852811 |
| MTR9763 | Mamanguape | 11 | -6.72091 | -35.17918 | KM852809 |
| MTR9765 | Mamanguape | 11 | -6.72091 | -35.17918 | KM852817 |
| MTR9766 | Mamanguape | 11 | -6.72091 | -35.17918 | KM852758 |
| MTR9767 | Mamanguape | 11 | -6.72091 | -35.17918 | KM852800 |
| MTR9768 | Mamanguape | 11 | -6.72091 | -35.17918 | KM852807 |
| MTR9769 | Mamanguape | 11 | -6.72091 | -35.17918 | KM852760 |
| JM165 | Manguaguape | 11 | -6.72091 | -35.17918 | KM852810 |
| JM166 | Manguaguape | 11 | -6.72091 | -35.17918 | KM852777 |
| JM167 | Manguaguape | 11 | -6.72091 | -35.17918 | KM852774 |
| MZUSP78149 | Maragogipe |  | -12.85915 | -38.86343 |  |
| 117 | Mata de São Jõao |  | -12.57489 | -38.04134 |  |
| RPD053 | Miguel Calmon | 24 | -11.3676 | -40.52073 | KM852744 |
| RPD062 | Miguel Calmon | 24 | -11.3927 | -40.53882 | KM852743 |
| RPD064 | Miguel Calmon | 24 | -11.3927 | -40.53882 | KM852753 |
| RPD112 | Miguel Calmon | 24 | -11.3664 | -40.52148 | KM852793 |
| RPD141 | Miguel Calmon | 24 | -11.3676 | -40.52073 | KM852806 |
| RPD200 | Miguel Calmon | 24 | -11.39453 | -40.53298 |  |
| MZUSP94882 | Miracema do Tocantins |  | -9.731 | -48.3867 |  |
| MTR22320 | Morro do Chapéu | 18 | -11.59195 | -41.20845 | KM852829 |
| MTR22321 | Morro do Chapéu | 18 | -11.59195 | -41.20845 | KM852827 |
| MTR22322 | Morro do Chapéu | 18 | -11.59195 | -41.20845 | KM852784 |
| MTR22577 | Morro do Chapéu | 18 | -11.59195 | -41.20845 | KM852828 |
| MTR10368 | Murici | 19 | -9.26389 | -35.93028 | KM852814 |
| MTR10369 | Murici | 19 | -9.26389 | -35.93028 | KM852805 |
| MTR17798 | Murici | 19 | -9.26389 | -35.93028 | KM852780 |
| MTR17817 | Murici | 19 | -9.26389 | -35.93028 | KM852822 |
| MTR09907 | Natal | 12 | -5.72211 | -35.22234 | KM852796 |
| MZUSP92287 | Olivença | 2 | -14.95 | -39.016 |  |
| MTR09770 | Olivença | 2 | -15.683 | -38.933 | KM852789 |
| MRT094 | Pacoti | 10 | -4.23028 | -38.91722 | KM852761 |
| MRT150 | Pacoti | 10 | -4.23028 | -38.91722 | KM852823 |
| MTR04534 | Pacoti | 10 | -4.23028 | -38.91722 | KM852767 |
| MTR04538 | Pacoti | 10 | -4.23028 | -38.91722 | KM852794 |
| ESTR0481 | Palmeiras do Tocantins |  | -6.67 | -47.53 |  |
| MPEG19030 | Paragominas |  | -3.074 | -47.334 |  |
| MZUSP94113 | Passo de Camaragibe |  | -9.233 | -35.483 |  |
| MNRJ11403 | Pilar |  | -9.58 | -35.955 |  |

| MTR09757 | Praia do Forte | 7 | -12.56913 | -38.0062 | KM852782 |
| --- | --- | --- | --- | --- | --- |
| MTR09758 | Praia do Forte | 7 | -12.56913 | -38.0062 | KM852778 |
| PEU071 | Presidente Trancredo Neves |  | -13.4115 | -39.299 |  |
| MZUSP36700 | Recife |  | -7.87058 | -35.0749 |  |
| MPEG24852 | Riachão |  | -7.351 | -46.536 |  |
| MZUSP23127 | Rio Formoso |  | -8.66798 | -35.14168 |  |
| MZUSP65669 | Rio Formoso |  | -8.72178 | -35.17286 |  |
| RPD365 | Salvador | 9 | -12.97125 | -38.44893 | KM852799 |
| RPD366 | Salvador | 9 | -12.97125 | -38.44893 | KM852790 |
| RPD367 | Salvador | 9 | -12.97125 | -38.44893 | KM852779 |
| RPD368 | Salvador | 9 | -12.97125 | -38.44893 | KM852791 |
| RPD369 | Salvador | 9 | -12.97125 | -38.44893 | KM852750 |
| RPD370 | Salvador | 9 | -12.97125 | -38.44893 | KM852763 |
| MZUSP88090 | Santa Luzia do Itanhy |  | -11.31546 | -37.53144 |  |
| MTR17850 | São Desidério |  | -12.326469 | -44.975202 | KM852765 |
| MTR17914 | São Desidério |  | -12.326469 | -44.975202 | KM852812 |
| MTR17915 | São Desidério |  | -12.326469 | -44.975202 | KM852815 |
| CHUNB 43897 | São Domingos |  | -13.59072 | -46.3901 |  |
| MZUSP65605 | São José dos Ramos |  | -7.252 | -35.384 |  |
| CX170/P18 | São Lourenço da Mata | 25 | -7.91633 | -35.02938 | KM852825 |
| MZUSP351 | São Miguel |  | -9.783 | -36.083 |  |
| MZUSP351 | São Miguel dos Campos |  | -9.783 | -36.083 |  |
| MTR25008 | Senhor do Bonfim |  | -10.55252 | -40.14507 |  |
| MZUSP59060 | Timbaúba |  | -7.5043 | -35.33586 |  |
| MD1721 | Una | 3 | -15.18375 | -39.07643 | KM852776 |
| MD2613 | Una | 3 | -15.18375 | -39.07643 | KM852826 |
| MTR16696 | Una | 3 | -15.28144 | -39.01637 | KM852786 |
| MD3287 | Una-Olivença | 2/3 | unavailable | unavailable | KM852746 |
| MD3288 | Una-Olivença | 2/3 | unavailable | unavailable | KM852773 |
| MD3224 | Una-Olivença | 2/3 | unavailable | unavailable | KM852795 |
| MD3204 | Una-Olivença | 2/3 | unavailable | unavailable | KM852742 |
| MTR09885 | Uruçuca | 4 | -14.5833 | -39.2833 | KM852797 |
| MTR09886 | Uruçuca | 4 | -14.5833 | -39.2833 | KM852762 |
| MTR09905 | Utinga |  | -11.92123 | -41.08248 |  |
| MZUSP78148 | Valença |  | -13.36143 | -39.08392 |  |
| MZUSP23105 | Vicência |  | -7.667 | -35.33 |  |
| MTR21983 | Wenceslau Guimarães | 26 | -13.5806 | -39.70467 | KM852748 |
| MTR22025 | Wenceslau Guimarães | 26 | -13.5806 | -39.70467 | KM852818 |
| MTR22268 | Wenceslau Guimarães | 26 | -13.5806 | -39.70467 | KM852783 |
| MTR22318 | Wenceslau Guimarães | 26 | -13.5806 | -39.70467 | KM852770 |
| MTR22319 | Wenceslau Guimarães | 26 | -13.5806 | -39.70467 | KM852772 |
| PEU012 | Wenceslau Guimarães | 26 | -13.56442 | -39.71 | KM852813 |
| PEU079 | Wenceslau Guimarães | 26 | -13.55927 | -39.72445 | KM852808 |

**Supplementary Table 2.** GenBank sequences used in phylogeographic analyses.

| GenBank accession number | Field Number | Species |
| --- | --- | --- |
| GQ140415 | MTR10109 | *Chatogekko amazonicus* |
| GQ140411 | MTR10186 | *Chatogekko amazonicus* |
| DQ110564 | JM207 | *Coleodactylus natalensis* |
| DQ104127 | JM207 | *Coleodactylus natalensis* |
| DQ104132 | MTR09791 | *Coloedactylus septentrionalis* |
| DQ104131 | MTR09795 | *Coloedactylus septentrionalis* |
| DQ104130 | MTR09789 | *Coloedactylus septentrionalis* |
| DQ110555 | MTR09792 | *Coloedactylus septentrionalis* |
| DQ110554 | MTR09794 | *Coloedactylus septentrionalis* |
| DQ110553 | MTR09793 | *Coloedactylus septentrionalis* |
| EU435278 | LG1177 | *Gonatodes humeralis* |
| EU435277 | Unavailable | *Lepidoblepharis xanthostigma* |
| EU435276 | MTR09894 | *Pseudogonatodes guianensis* |
| EU435275 | MTR09893 | *Pseudogonatodes guianensis* |

**Supplementary Table 2.** Thermal physiology data from experiments after capture and historical climatic stability and current climate data from localities (Tpref = preferred temperature, CTmax = critical thermal maximum, CTmin = critical thermal minimum, Ttol = thermal tolerance, calculated as CTmax – CTmin, season = season when experiment was performed, Residual Ttol = Ttol residuals after controlling the effect of season, Temp. season. = temperature seasonality (=BIO4, standard deviation of temperature over the year), Ann. temp. range = annual temperature range (=BIO5 – BIO6)).

| Sample | Locality | Stability | Tpref | CTmax | CTmin | Ttol | Season | Residual Ttol | Temp. season. | Ann. temp. range (^o^C) |
| --- | --- | --- | --- | --- | --- | --- | --- | --- | --- | --- |
| RPD365 | Salvador | 0.7393 | 20.1 | 40.75 | 16.62 | 24.13 | Winter | -2.8954545 | 1.118 | 10.0 |
| RPD366 | Salvador | 0.7393 | 31.1 | 40.16 | 13.29 | 26.87 | Winter | -0.1554545 | 1.118 | 10.0 |
| RPD367 | Salvador | 0.7393 | 25.4 | 41.63 | 17.78 | 23.85 | Winter | -3.1754545 | 1.118 | 10.0 |
| RPD368 | Salvador | 0.7393 | 19.2 | 41.7 | 15.3 | 26.4 | Winter | -0.6254545 | 1.118 | 10.0 |
| RPD369 | Salvador | 0.7393 | 26.3 | 43.36 | 18.1 | 25.26 | Winter | -1.7654545 | 1.118 | 10.0 |
| RPD370 | Salvador | 0.7393 | 24.05 | 37.6 | 15.1 | 22.5 | Winter | -4.5254545 | 1.118 | 10.0 |
| MTR22320 | Morro do Chapéu | 0.37483 | 28.55 | 37.93 | 15.03 | 22.9 | Summer | -0.1857143 | 1.345 | 13.5 |
| MTR22322 | Morro do Chapéu | 0.37483 | 22.4 | - | 18.4 | - | Summer | - | 1.345 | 13.5 |
| MTR22577 | Morro do Chapéu | 0.37483 | 24.35 | 36.25 | 18.8 | 17.45 | Summer | -5.6357143 | 1.345 | 13.5 |
| RPD053 | Miguel Calmon | 0.50297 | - | 40.77 | 12.13 | 28.64 | Winter | 1.6145455 | 1.506 | 14.2 |
| RPD062 | Miguel Calmon | 0.50297 | - | 41.15 | 11.73 | 29.42 | Winter | 2.3945455 | 1.506 | 14.2 |
| RPD064 | Miguel Calmon | 0.50297 | - | 42.3 | 7.37 | 34.93 | Winter | 7.9045455 | 1.506 | 14.2 |
| RPD112 | Miguel Calmon | 0.50297 | - | 40.21 | 11.04 | 29.17 | Winter | 2.1445455 | 1.506 | 14.2 |
| RPD141 | Miguel Calmon | 0.50297 | - | 39.31 | 13.2 | 26.11 | Winter | -0.9154545 | 1.506 | 14.2 |
| MTR22318 | Wenceslau Guimarães | 0.61296 | 24.5 | 38.12 | 14.58 | 23.54 | Summer | 0.4542857 | 1.272 | 11.0 |
| MTR22319 | Wenceslau Guimarães | 0.61296 | 22.6 | 37.4 | 17.3 | 20.1 | Summer | -2.9857143 | 1.272 | 11.0 |
| MTR24519 | Campo Formoso | 0.63213 | - | 39.02 | 12.46 | 26.56 | Summer | 3.4742857 | 1.491 | 14.7 |
| MTR24520 | Campo Formoso | 0.63213 | - | 36.5 | 19.9 | 16.6 | Summer | -6.4857143 | 1.491 | 14.7 |
| MTR24521 | Campo Formoso | 0.63213 | - | 37.29 | 21.59 | 15.7 | Summer | -7.3857143 | 1.491 | 14.7 |
| MTR19913 | Chapada | 0.65173 | 25.6 | 38.24 | 16.86 | 21.38 | Summer | -1.7057143 | 1.232 | 15.5 |
| MTR19914 | Chapada | 0.65173 | 22.9 | 39.93 | 13.22 | 26.71 | Summer | 3.6242857 | 1.232 | 15.5 |
| RPD130 | Chapada | 0.65173 | 24 | 42.42 | 14.53 | 27.89 | Summer | 4.8042857 | 1.232 | 15.5 |
| RPD133 | Chapada | 0.65173 | 21 | 42.72 | 15.46 | 27.26 | Summer | 4.1742857 | 1.232 | 15.5 |
| MTR19942 | Chapada | 0.65173 | 25.8 | 41.19 | 16.17 | 25.02 | Summer | 1.9342857 | 1.232 | 15.5 |
| MTR19944 | Chapada | 0.65173 | 26.3 | 41.56 | 14.37 | 27.19 | Summer | 4.1042857 | 1.232 | 15.5 |

**Supplementary Table 3.** Thermal physiology data from experiments after acclimation treatments (Tpref = preferred temperature, CTmax = critical thermal maximum, CTmin = critical thermal minimum, Ttol = thermal tolerance, calculated as CTmax – CTmin).

| Acclimation treatment “cool” | | | | | | | |
| --- | --- | --- | --- | --- | --- | --- | --- |
| Sample | Locality | Average acclimation temperature (^o^C) | Acclimation duration (days) | Tpref (^o^C) | CTmax (^o^C) | CTmin (^o^C) | Ttol (^o^C) |
| RPD053 | Miguel Calmon | 20.76 | 14 | 28.1 | 43.9 | 13.12 | 30.78 |
| RPD062 | Miguel Calmon | 20.76 | 14 | 18.6 | 43.42 | 12.33 | 31.09 |
| RPD064 | Miguel Calmon | 20.76 | 14 | 26.7 | 46.13 | 12.24 | 33.89 |
| RPD112 | Miguel Calmon | 20.76 | 14 | 23.55 | 45.63 | 12.49 | 33.14 |
| RPD141 | Miguel Calmon | 20.76 | 14 | 32.4 | 41.07 | 12.5 | 28.57 |
| MTR19914 | Chapada | 23.91 | 11 | 22.2 | 42.7 | 12.5 | 30.2 |
| MTR19942 | Chapada | 23.91 | 12 | 20 | 38.38 | 14.77 | 23.61 |
| RPD130 | Chapada | 22.66 | 14 | 21 | 41.05 | 13.75 | 27.3 |
| RPD133 | Chapada | 22.06 | 18 | 24.5 | 41.88 | 14.27 | 27.61 |
| C12P61 | Catu | 20.76 | 13 | 24.3 | 41.96 | 8.56 | 33.4 |
| C3P21 | Catu | 20.76 | 14 | 22.1 | 40.23 | 11.53 | 28.7 |
| Frag11P87 | Catu | 20.76 | 14 | 28.05 | 41.85 | 8.79 | 33.06 |
| Frag12P63 | Catu | 20.76 | 11 | 23.95 | 42.81 | 8.64 | 34.17 |
| P29 | Catu | 20.76 | 14 | 26.95 | 41.5 | 11.5 | 30 |
| P69C12 | Catu | 20.76 | 13 | 22 | 41.75 | 11.55 | 30.2 |
| PC27 | Catu | 20.76 | 14 | 25.1 | 43.23 | 11.22 | 32.01 |
| Acclimation treatment “warm” | | | | | | | |
| Sample | Locality | Average acclimation temperature (^o^C) | Acclimation duration (days) | Tpref (^o^C) | CTmax (^o^C) | CTmin (^o^C) | Ttol (^o^C) |
| RPD062 | Miguel Calmon | 27.93 | 14 | 26 | 43.05 | 10.21 | 32.84 |
| RPD064 | Miguel Calmon | 27.93 | 14 | 20.25 | 43.77 | 11.65 | 32.12 |
| RPD112 | Miguel Calmon | 27.93 | 14 | 13.05 | 41.65 | 13.22 | 28.43 |
| RPD141 | Miguel Calmon | 28.68 | 18 | 24.8 | 37.4 | 16.63 | 20.77 |
| MTR19944 | Chapada | 30.17 | 12 | 22.2 | 41.87 | 18.13 | 23.74 |
| RPD133 | Chapada | 30.43 | 14 | 31.05 | 41.99 | 18.3 | 23.69 |
| C12P61 | Catu | 28.18 | 14 | 21.65 | - | 11.55 | - |
| C3P21 | Catu | 27.93 | 14 | 28.5 | 42.95 | 11.27 | 31.68 |
| Frag11P87 | Catu | 28.18 | 15 | 25.6 | 43.27 | 12.3 | 30.97 |
| P29 | Catu | 28.18 | 14 | 19.2 | 43.72 | 12.03 | 31.69 |
| P69C12 | Catu | 28.18 | 14 | 18.05 | 42.33 | 12.63 | 29.7 |
| Extra acclimation treatment | | | | | | | |
| Sample | Locality | Average acclimation temperature (^o^C) | Acclimation duration (days) | Tpref (^o^C) | CTmax (^o^C) | CTmin (^o^C) | Ttol (^o^C) |
| RPD053 | Miguel Calmon | 20.77 | 5 | 23.05 | - | - | - |
| RPD062 | Miguel Calmon | 20.77 | 5 | 23.5 | - | - | - |
| RPD064 | Miguel Calmon | 20.77 | 7 | 22 | - | - | - |
| RPD112 | Miguel Calmon | 20.77 | 7 | 25 | - | - | - |
| C12P61 | Catu | 26.43 | 27 | 20.4 | 42.71 | 9.87 | 32.84 |
| C3P21 | Catu | 25.68 | 20 | 21.4 | 42.71 | 9.41 | 33.3 |
| Frag11P87 | Catu | 26.18 | 25 | 25.7 | 41.92 | 10.29 | 31.63 |
| Frag12P63 | Catu | 25.68 | 38 | 24.2 | 44.55 | 11.63 | 32.92 |
| P29 | Catu | 25.93 | 29 | 21 | 43.51 | 11.73 | 31.78 |
| P69C12 | Catu | 26.43 | 32 | 29.75 | 44.7 | 11.34 | 33.36 |
| PC27 | Catu | 26.18 | 25 | 22.4 | 41.13 | 10.24 | 30.89 |

1. **References**

Aljanabi, S. M., and Martinez, I. (1997). Universal and rapid salt-extraction of high quality genomic DNA for PCR-based techniques. *Nucleic acids research* 25, 4692–4693.

Brooks, G. R., and Sassaman, J. F. (1965). Critical thermal maxima of larval and adult *Eurycea bislineata*. *Copeia*, 251–252.

Carnaval, A. C. C., Waltari, E., Rodrigues, M. T., Rosauer, D., VanDerWal, J., Damasceno, R., et al. (2014). Prediction of phylogeographic endemism in an environmentally complex biome. *Proc. R. Soc. B Biol. Sci.* 281, 1471–2954. doi: 10.1098/rspb.2014.1461

Cowles, R. B., and Bogert, C. M. (1944). A preliminary study of the thermal requirements of desert reptiles. *Iguana* 83, 53.

Gamble, T., Daza, J. D., Colli, G. R., Vitt, L. J., and Bauer, A. M. (2011). A new genus of miniaturized and pug‐nosed gecko from South America (Sphaerodactylidae: Gekkota). *Zool. J. Linnean Soc.* 163, 1244–1266.

Geurgas, S. R., Rodrigues, M. T., and Moritz, C. (2008). The genus *Coleodactylus* (Sphaerodactylinae, Gekkota) revisited: A molecular phylogenetic perspective. *Mol. Phylogen. Evol.* 49, 92–101.

Hijmans, R.J., S.E. Cameron, J.L. Parra, P.G. Jones and Jarvis, A. (2005). Very high resolution interpolated climate surfaces for global land areas. *Int. J. Climatol.* 25, 1965-1978.

Stamatakis, A. (2006). RAxML-VI-HPC: maximum likelihood-based phylogenetic analyses with thousands of taxa and mixed models. *Bioinformatics* 22, 2688–2690.

R Development Core Team (2008). R: A language and environment for statistical computing. R Foundation for Statistical Computing, Vienna, Austria. ISBN 3-900051-07-0, URL http://www.R-project.org.

Whiting, A. S., Bauer, A. M., and Sites, J. W., Jr (2003). Phylogenetic relationships and limb loss in sub-Saharan African scincine lizards (Squamata: Scincidae). *Mol. Phylogen. Evol.* 29, 582–598.
